# Oncolytic Viruses in Cancer Immunotherapy: From Molecular Engineering to Clinical Translation

**DOI:** 10.3390/cells15050393

**Published:** 2026-02-24

**Authors:** Mohammad Fayyad-Kazan, Sarah Al-Tameemi, Allal Ouhtit

**Affiliations:** 1 Department of Natural and Applied Sciences, College of Arts and Sciences, American University of Iraq-Baghdad (AUIB), Baghdad 10011, Iraq; sarah.majeed@auib.edu.iq; 2 College of Health Sciences, American University of Iraq-Baghdad (AUIB), Baghdad 10011, Iraq

**Keywords:** oncolytic virus, immunotherapy, cancer, tumor microenvironment, combination therapy, immunogenic cell death

## Abstract

**Highlights:**

**What are the main findings?**

- Oncolytic viruses exert antitumor activity through direct tumor lysis and induction of systemic antitumor immunity.
- Advanced viral engineering strategies enhance tumor selectivity, immune activation, and therapeutic efficacy.

**What are the implications of the main findings?**

- Rational combination therapies are critical to overcome antiviral immunity and tumor resistance.
- The findings guide the development of next-generation oncolytic virus platforms and clinical trial design.

**Abstract:**

Cancer immunotherapy has transformed modern oncology, yet durable responses remain limited for many patients due to immune exclusion, adaptive resistance, and tumor heterogeneity. Oncolytic viruses (OVs) have emerged as a novel class of immunotherapeutics that unify direct tumor cytolysis with stimulation of antitumor immunity. By inducing immunogenic cell death (ICD) and releasing tumor-associated antigens (TAAs), OVs remodel the tumor microenvironment (TME) into an inflamed and immune-permissive niche capable of enabling systemic immune activation. Rapid advances in viral engineering have strengthened the translational potential of OVs through tumor-selective gene deletions, tumor-specific promoters, microRNA-based detargeting, and receptor-retargeting strategies that collectively enhance safety, specificity, and intratumoral propagation. Next-generation OVs are increasingly “armed” with immunostimulatory payloads—including cytokines, chemokines, checkpoint inhibitors, bispecific T-cell engagers, and suicide gene systems—allowing localized immune modulation with reduced systemic toxicity. These innovations have propelled significant clinical progress, exemplified by the approvals of talimogene laherparepvec (T-VEC), G47Δ, and H101, and have driven a surge of combination trials integrating OVs with immune checkpoint blockade, adoptive cell therapies, radiotherapy, and targeted therapies to overcome multilayered tumor immune resistance. Despite this momentum, clinical implementation remains challenged by antiviral immunity, heterogeneous viral distribution, stromal barriers, and dynamic interferon (IFN) signaling in the TME. Emerging delivery approaches, including carrier cell systems, nanotechnology-enabled viral shielding, and synthetic virology platforms, offer promising solutions to these limitations. Oncolytic virotherapy is rapidly evolving into a multifunctional immunotherapeutic platform capable of reshaping antitumor responses at both local and systemic levels. By integrating advanced viral engineering with rational combination strategies and innovative delivery technologies, OVs hold substantial potential to overcome current barriers in cancer immunotherapy and advance precision oncology. Continued translational research will be essential to fully harness their therapeutic impact and broaden their clinical applicability.

## 1. Introduction

Cancer remains a leading cause of mortality worldwide, underscoring the urgent need for innovative therapeutic strategies. Traditional treatments such as chemotherapy and radiotherapy, while effective, often lack specificity and can lead to significant side effects. In contrast, immunotherapy, which harnesses the body’s own immune system to fight cancer, has revolutionized treatment outcomes for many patients. Among novel immunotherapeutic approaches, oncolytic virotherapy stands out as a promising modality that merges direct cytotoxicity with immunomodulation [1].

The concept of using viruses to treat cancer dates back to observations of spontaneous tumor regression following viral infections. This led to the development of the first genetically engineered OV, ONYX-015, a replication-selective adenovirus designed to target p53-deficient tumors [2]. Building upon these foundations, the field has advanced significantly, culminating in the approval of more sophisticated agents like Talimogene Laherparepvec (T-VEC) and the development of next-generation viruses such as G47Δ, which incorporate advanced genetic engineering for enhanced selectivity and immune stimulation [3].

OVs are either engineered or naturally occurring viruses that selectively replicate within and lyse tumor cells, sparing normal tissue. Selectivity is achieved by exploiting the inherent defects in tumor cell signaling pathways, such as the p53 pathway, or by engineering the virus to express therapeutic transgenes under tumor-specific promoters. The direct lysis of tumor cells releases (TAAs) and damage-associated molecular patterns (DAMPs), initiating a cascade of immune activation. This process, known as ICD, can transform an immunologically “cold” tumor into a “hot” one, characterized by increased immune cell infiltration and activation [4].

The dual mechanism of action—direct oncolysis and immune stimulation—positions OVs as ideal candidates for combination therapies. Synergistic benefits have been observed when OVs are combined with ICIs, which block inhibitory signals (e.g., PD-1/PD-L1, CTLA-4) and unleash pre-existing anti-tumor T cells, or with ACTs like CAR-T cells, which introduce engineered immune cells targeting specific tumor antigens [5]. Moreover, combining OVs with conventional treatments, reduce tumor burden, and overcome resistance mechanisms.

This review provides a comprehensive overview of recent advances in OV therapy, particularly focusing on their integration with immunotherapeutic strategies. We discuss the mechanisms of action, highlight key clinical developments, discuss engineering strategies for enhanced efficacy, and explore the challenges and future directions in this rapidly evolving field.

## 2. Mechanisms of Action: Direct Oncolysis and Immune Stimulation

**Figure 1:**
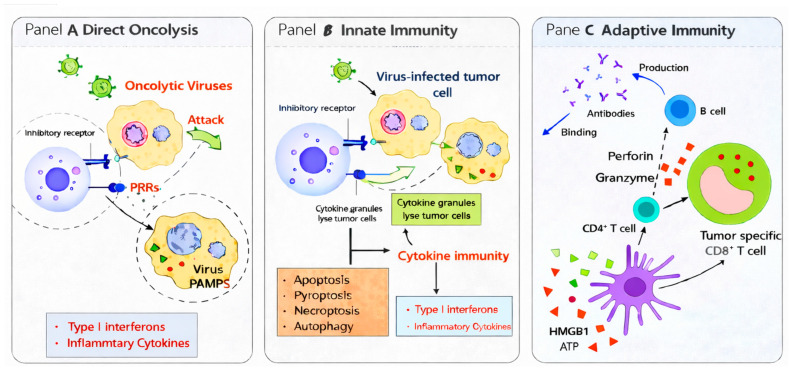
**OV-induced immunogenic cell death (ICD) and coordinated activation of innate and adaptive antitumor immunity. Panel** (**A**): Direct Oncolysis. OVs selectively infect tumor cells and replicate intracellularly, leading to direct tumor cell lysis. Viral replication generates PAMPs that are sensed by PRRs, triggering the production of type I IFNs and inflammatory cytokines. OV-mediated lysis further enhances tumor antigen release and contributes to local immune activation. **Panel** (**B**): These signals activate innate immune pathways and promote cytokine-driven immunity. NK cells recognize virus-infected tumor cells through the integration of activating and inhibitory receptor signals and contribute to early cytotoxic clearance via cytokine release and granule-mediated tumor cell lysis. **Panel** (**C**): cytotoxic T lymphocytes. Activated CD8^+^ T cells eliminate tumor cells through perforin- and granzyme-mediated killing, while CD4^+^ T cells provide essential helper functions to sustain and amplify the antitumor response. In parallel, B cells recognize viral antigens and produce antibodies, reinforcing both antiviral and antitumor immunity. Collectively, these adaptive immune mechanisms generate a durable and systemic antitumor response targeting both virus-infected and uninfected tumor cells.

The therapeutic efficacy of OVs is mediated through two primary, interconnected mechanisms: direct tumor cell lysis and the induction of anti-tumor immune responses (Figure 1).

OVs preferentially infect and replicate within tumor cells owing to intrinsic defects in antiviral signaling pathways and dysregulated oncogenic signaling that render the cancer cell environment permissive for viral propagation. A hallmark example is the impairment of the type I IFN pathway, a central antiviral defense system. As shown in several vitro, in vivo and clinical studies, many tumors exhibit alterations in IFN signaling pathways, including components of the JAK/STAT axis (e.g., STAT1, STAT2), which may involve context-dependent gain- or loss-of-function events that ultimately shape antiviral responsiveness and immune evasion. These deficiencies suppress the induction of IFN-stimulated genes (ISGs) such as PKR, OAS1, and MxA, which normally block viral replication, thereby allowing OVs to replicate unrestrained [6]. Beyond IFN signaling, an in vivo study reported that tumor cells often harbor aberrant RAS/MAPK activation, a feature that enhances permissiveness to OVs such as reoviruses, which rely on hyperactive RAS signaling for efficient uncoating and replication [7]. Similarly, an in vitro study reported that dysregulation of EGFR signaling and overexpression of HER2/neu have been shown to support adenoviral replication by augmenting E2F-driven transcription of viral genes [8]. Another in vitro and in vivo observations indicated that critical determinant is the loss of tumor suppressors. Deficiency in p53, which normally induces apoptosis in virally infected cells, allows tumor cells to survive long enough to support multiple viral replication cycles [9]. In addition, deletion of PTEN (Phosphatase and TENsin homolog), in vivo, removes its inhibitory brake on the PI3K/AKT/mTOR pathway, resulting in elevated nucleotide pools, enhanced glycolysis, and global increases in protein synthesis—conditions that not only fuel uncontrolled tumor proliferation but also favor viral genome replication and structural protein production [10]. Furthermore, the in vitro and in vivo deregulation of the Rb/E2F axis, common in many cancers, creates a replication-permissive environment for DNA viruses such as adenoviruses. For instance, mutant adenoviruses like ONYX-015 are engineered to replicate selectively in Rb-deficient cells, exploiting this vulnerability [11]. Similarly, in vitro, in vivo, and emerging clinical evidences highlighted that HSV mutants lacking the γ34.5 gene replicate more efficiently in tumor cells with defective PKR-mediated translational shutoff, while sparing normal cells with intact antiviral control [12]. Taken together, these molecular alterations, ranging from defective IFN responses and tumor suppressor loss to oncogenic signaling hyperactivation, synergize to transform malignant cells into “viral factories.” This selective vulnerability underlies the therapeutic window of OVs and explains why they preferentially amplify within tumors while being attenuated in healthy tissues.

Immune Stimulation and TME Remodeling. OVs not only destroy tumor cells through direct lysis but also profoundly reshape the TME and convert tumors into in situ personalized vaccines by stimulating innate and adaptive immunity. Viral oncolysis causes the release of TAAs, DAMPs such as calreticulin, HMGB1, ATP, and pathogen-associated molecular patterns (PAMPs) derived from viral genomes and proteins [13]. Together, these molecules signal ICD, which is essential for converting cell death into an immune-activating event. The DAMPs and PAMPs activate antigen-presenting cells (APCs), especially DCs, which engulf and process TAAs. Processed antigens are then presented on MHC class I and II molecules to T lymphocytes, priming both CD4^+^ helper T cells and CD8^+^ cytotoxic T lymphocytes (CTLs), thereby initiating robust adaptive anti-tumor immunity. This fundamental characteristic explains why non-injected lesions can regress: priming is systemic, not restricted to the directly infected primary tumor site. On this basis, several in vitro and in vivo mouse models indicated that a major design direction in next-generation OVs involves encoding cytokines and ligands such as GM-CSF, IL-12, and CD40L which fortify APC licensing, or chemokines such as CXCL9/10 which improve lymphocyte trafficking. OV genomes and replication intermediates activate multiple pattern-recognition receptors (PRRs), including TLR3, RIG-I, MDA5, and the cGAS–STING cytosolic DNA-sensing pathway. Which PRR axis predominates during OV infection determines the balance, potency, and timing of type I IFN and pro-inflammatory cytokines that mature DCs, especially Batf3^+^ cross-presenting cDC1 cells, and ultimately gate CTL priming against tumor epitopes [14]. OVs or payloads that robustly activate STING/IRF3 generate polyfunctional, stem-like CD8^+^ T cells and cooperate synergistically with immune checkpoint blockade. Conversely, tumors harboring intrinsic defects in cGAS/STING may particularly benefit from OV strategies that deliver these signals exogenously [14]. Therefore, a key OV engineering principle is to avoid viral genes that antagonize STING/IFN in immune cells and, instead, consider encoding STING agonists or RIG-I ligands to ensure an early, sharp IFN pulse that licenses cross-priming without chronically exhausting T cells [14].

OVs trigger multiple regulated cell death (RCD) programs including apoptosis, necroptosis, pyroptosis, and sometimes autophagy-associated lysis. These death modalities are not immunologically interchangeable. Necroptosis and pyroptosis more readily expose calreticulin and release ATP, HMGB1, and IL-1 family cytokines—hallmarks of ICD that increase cDC maturation and favor Th1 skewing [15]. Engineering OVs to delete their viral anti-apoptotic genes or to express TRAIL, IL-12, or other pathway modulators that tip death toward necroptosis/pyroptosis significantly increases cross-priming and enhances bystander killing in non-infected tumor areas [15]. Payloads that push MLKL/gasdermin executioner pathways can convert otherwise “silent” death into ICD, and these should ideally be combined with DC-licensing transgenes to convert ICD into stronger T cell priming [15].

Batf3^+^ cDC1 cells are the primary cross-presenters of cell-associated tumor antigens, and OV-driven IFN and CD40 signaling upregulate antigen-processing machinery (TAP1/2, immunoproteasome subunits) and costimulatory molecules in these cells. This enables efficient cross-presentation not only in draining lymph nodes, but also later in tertiary lymphoid structures (TLS). The balance between endosome-to-cytosol cross-presentation versus vacuolar cross-presentation is influenced by inflammatory tone and the density and type of DAMPs/PAMPs generated by the OV. Expanding cDC1 pools using Flt3L, or licensing them with CD40 agonists, synergizes strongly with OV activity [16].

Following OV priming in this inflammatory milieu, several clinical and preclinical immunotherapy studies showed that T-cell responses frequently “spread” from initial viral/transgene epitopes or dominant tumor epitopes to subclonal or cryptic tumor epitopes, a process known as epitope spreading, enabling T-cell-mediated killing of non-infected tumor regions [17]. Timing is critical; a short, early type I IFN burst supports effector programming, while prolonged IFN exposure increases PD-L1, upregulates IDO, and accelerates exhaustion. These dynamics suggest a rational sequencing principle: checkpoint blockade with PD-1/PD-L1 ± CTLA-4 is optimally applied after the initial priming window [18].

Several in vivo and ex vivo immune models revealed that OVs also directly activate NK cells through missing-self recognition, NKG2D ligands, and cytokines such as IL-12, IL-15, and type I IFNs. NK-derived IFN-γ and contact-dependent signals enhance DC recruitment and maturation, bridging innate and adaptive responses [19]. As antiviral or anti-transgene antibodies arise, antibody-dependent cellular cytotoxicity (ADCC) and antibody-dependent cellular phagocytosis (ADCP) can aid clearance of infected tumor cells; thus, humoral immunity is not purely antagonistic once priming has been achieved. Moreover, encoding IL-15/IL-15Rα complexes, or engineering OVs to avoid excessive restoration of MHC-I on tumor cells, can amplify NK-mediated early control [19].

In vivo tumor models and early clinical trials revealed that PRR/IFN signaling can reprogram immunosuppressive myeloid populations such as TAMs and MDSCs toward antigen-presenting, inflammatory states characterized by increased IL-12 and CXCL9/10 and reduced arginase-1, normalize tumor vasculature, and reduce interstitial fluid pressure, together enabling T cell infiltration and drug access. Recent oncolytic HSV data demonstrate TME-wide reprogramming, including MHC-II upregulation on residual tumor cells, emphasizing that immune benefits from OVs outlast direct oncolysis [20]. Rational OV combinations pairing CSF1R blockade or PI3Kγ inhibition can “lock in” TAMs into inflammatory states, and arming OVs with TGF-β traps or CXCL9/10 can enhance effector T-cell recruitment and retention [20].

Immunocompetent mouse tumor models and primary human tumor samples showed that OV-induced IFN also restores MHC-I antigen presentation components in tumor cells with soft immune escape, re-sensitizing them to CTL-mediated killing [21]. In contrast, lesions with hard antigen-presentation defects, such as B2M or JAK1 loss-of-function, may respond better to OVs engineered to favor NK-dominant elimination or express bispecific engagers that allow T or NK cells to target tumor antigens independently of MHC-I [21].

In vivo mouse models and human clinical correlations reported that sustained yet organized OV-driven inflammation combined with lymphoid chemokines (CCL19/21, CXCL13) can nucleate tertiary lymphoid structures (TLS) within tumors, enabling on-site B and T cell priming and affinity maturation, correlates of breadth and durability of immune control [22]. Certain viral backbones such as HSV and vaccinia appear especially competent at TLS induction when combined with appropriate cytokine payloads.

In vivo and ex vivo macrophage studies unraveled that short-lived epigenetic training of monocytes and macrophages following OV exposure (e.g., H3K4me3 and H3K27ac changes) can increase secondary responsiveness, creating a form of trained innate immunity [23]. Meanwhile, tumor metabolic rewiring (lactate, arginine, tryptophan metabolism) influences T/NK cell function and exhaustion. Combining OVs with IDO or arginase inhibitors and low-dose anti-VEGF to improve perfusion and vascular normalization may consolidate effector function [24].

At the spatial level, Immunocompetent mouse models with immune synapse analysis showed that OV infection is often patchy; however, immune control spreads beyond infected regions through CTL/NK trafficking and chemokine gradients. Strategically spaced intratumoral injections or vascular-tropic OV backbones can improve coverage, while epitope spreading and systemic T-cell mobilization explain tumor control at distant sites, especially when synchronized with checkpoint blockade [25].

Different OV backbones show characteristic immunobiological tendencies. Vaccinia-based OVs replicate rapidly, activate myeloid and NK compartments strongly, and can promote necroptosis; they are often used for payloading GM-CSF or IL-12. Adenoviruses have robust transgene payload capacity, induce apoptosis and autophagy-associated lysis, and allow precise tumor-selective promoter engineering [26]. Clinical data revealed that oncolytic HSV displays broad tropism, induces potent TME reprogramming, restores antigen presentation, and has already achieved clinical traction (e.g., T-VEC) [27]. Preclinical and clinical OV strategies reported that other platforms such as reovirus, VSV, measles virus, and NDV have distinct PRR footprints (especially RIG-I/MDA5 bias), making them excellent for IFN-rich licensing and in situ vaccination, provided safety engineering is carefully optimized [28]. Combination logic and dosing sequence are therefore essential. The most common rational sequence is OV at day 0, allow 2–7 days for the initial antigen/DAMP burst and cDC1 licensing, and then administer PD-1/PD-L1 ± CTLA-4 once activation markers increase but before exhaustion dominates [28]. Such combinations can be further supported by myeloid reprogrammers (CSF1R, PI3Kγ inhibitors), metabolic modulators (IDO/arginase inhibitors), and DC enhancers such as Flt3L or CD40 agonists; adoptive cell therapies such as ACT, TIL infusion, or CAR-T cells can then be layered “on top” of an OV-conditioned TME [28].

## 3. Enhancing OV Efficacy: Engineering and Combination Strategies

To maximize therapeutic potential, extensive research focuses on engineering OVs and combining them with other treatments. A major emerging design direction in OV development is the intentional promotion of ICD rather than silent apoptosis, thereby enhancing antitumor immune priming and therapeutic synergy.

### 3.1. Genetic Engineering for Tumor Selectivity and Potency

Viruses can be genetically modified to enhance their tumor specificity, reduce off-target effects, and increase their immunostimulatory potential. Common strategies include:

#### 3.1.1. Attenuation/Deletion

A foundational biotechnology strategy to achieve tumor selectivity in OVs is attenuation through targeted gene deletion, in which viral genes required for replication in normal cells are removed. These genetic modifications exploit the fact that tumor cells often harbor defective antiviral signaling pathways (e.g., impaired type I IFN, PKR, or p53 pathways), making them permissive to viral replication, while normal cells remain resistant [29]. A classical in vitro example is the deletion of the γ34.5 (ICP34.5) gene in Herpes Simplex Virus (HSV). This viral protein normally counteracts the host’s PKR–eIF2α pathway, which shuts down protein synthesis in response to infection. In γ34.5-deleted HSV, replication is blocked in normal cells with intact PKR signaling. However, in tumor cells where PKR is often defective, the virus remains replication-competent [30]. This approach underlies the design of Talimogene laherparepvec (T-VEC), the first FDA-approved OV, which combines γ34.5 deletion with insertion of GM-CSF to enhance immune stimulation [31]. In oncolytic adenoviruses, deletion of E1B-55K (e.g., in ONYX-015) using in vivo glioma models prevents inactivation of p53. Normal cells with functional p53 undergo apoptosis upon infection, limiting viral replication, while p53-deficient tumor cells allow full viral replication [32]. Using vivo immunodeficient as well as permissive models, it was shown that additional deletions in the E1A CR2 domain target Rb-defective tumor cells, creating conditional replication restricted to cancers with dysregulated Rb/E2F signaling [33]. Attenuated vaccinia strains are engineered by deleting thymidine kinase (TK) or vaccinia growth factor (VGF) genes. These enzymes were shown using vivo model to be critical for viral DNA replication in quiescent cells, but dispensable in rapidly proliferating tumor cells that already have elevated nucleotide synthesis and mitogenic signaling [34]. This ensures selective replication in malignant tissues.

Despite their success, gene-deletion approaches are not without limitations. First, attenuation often reduces viral fitness, resulting in lower replication kinetics and intratumoral spread, which can compromise oncolytic potency, especially in poorly vascularized or heterogenous tumors. For example, while γ34.5 deletion enhances HSV safety, it also diminishes replication efficiency and cytotoxicity in certain tumor subtypes. To mitigate this, newer HSV-based OVs (e.g., G47Δ) introduce compensatory mutations or promoter rewiring to partially restore replication competence while maintaining safety [35].

Second, tumor selectivity is not absolute. Many “tumor-specific” defects such as p53 or Rb inactivation are heterogeneous or reversible, and promoter or pathway leakiness can permit unintended replication in some normal proliferating cells, raising safety concerns in sensitive tissues [36]. The incomplete correlation between a molecular defect (e.g., p53 loss) and permissiveness to OV replication has been a recurring translational barrier.

Third, tumor evolution and immune clearance can undermine long-term efficacy. Attenuated viruses often elicit robust antiviral immune responses, leading to rapid clearance before full tumor lysis or immune priming occur. Furthermore, large-scale manufacturing of genetically modified viruses with multiple deletions or insertions can present stability and yield challenges, requiring stringent production control and regulatory oversight [37]. Hence, while attenuation and deletion strategies have been instrumental in establishing the safety profile of modern OVs, future designs must balance safety with replicative vigor, integrate adaptive control mechanisms (e.g., microRNA-targeted detargeting), and account for tumor heterogeneity to achieve consistent efficacy across diverse malignancies.

#### 3.1.2. Tumor-Specific Promoters

Another biotechnology strategy to enhance OV tumor selectivity is the use of tumor-specific or tumor-associated promoters to control the expression of essential viral genes or therapeutic transgenes. In this approach, viral replication and/or payload expression is placed under the regulation of promoters that are highly active in malignant tissues but silent or minimally active in normal cells. This ensures that viral replication is restricted to tumor cells, providing an additional layer of safety and specificity. A classic example is the use of the prostate-specific antigen (PSA) promoter or the prostate-specific membrane antigen (PSMA) enhancer/promoter to drive adenoviral E1A or E1B genes, thereby restricting viral replication to prostate cancer cells. This approach has been used in the design of CV706 and Ad-PSA, engineered adenoviruses that replicate selectively in PSA-expressing prostate tumors while sparing normal tissues [38]. Moreover, since the human telomerase reverse transcriptase (hTERT) promoter is strongly active in ~85–90% of tumors but not in most normal somatic cells, it is widely used to regulate OV replication. For example, adenoviruses such as OBP-301 (Telomelysin) with E1A under hTERT control replicate efficiently in telomerase-positive tumor cells, providing broad-spectrum tumor selectivity [39]. Further, Survivin, an inhibitor of apoptosis protein (IAP), is overexpressed in many cancers but absent in most differentiated tissues. Both in vitro tumor cells and in vivo xenograft models demonstrated that promoter-driven OVs such as Surv.m-CRAd utilize survivin promoters to regulate E1A expression, selectively replicating in survivin-high tumor cells [40]. Similar strategies employ BIRC5, BCL2, or MUC1 promoters to further enhance specificity in different tumor contexts. Given that many tumors exhibit hypoxic microenvironments, hypoxia-inducible factor (HIF)-responsive promoters (containing hypoxia-response elements, HREs) can be incorporated into OVs to activate viral replication in low-oxygen conditions. As demonstrated by Hypoxia-exposed cancer cell lines as well as mouse tumors, this allows viruses such as HRE-driven adenoviruses or vaccinia constructs to selectively thrive in hypoxic tumor cores, where normal tissue remains resistant [41]. Recent advances in synthetic promoter engineering and bioinformatics-guided design allow creation of hybrid promoters with enhanced tumor selectivity and transcriptional strength. For instance: Dual-regulatory promoters, combining tumor-selective and inducible elements (e.g., hTERT + HRE), restrict replication to both telomerase-positive and hypoxic tumor niches [42]. CRISPR/Cas9-based promoter editing allows precise insertion of tumor-specific promoter sequences into viral genomes [43]. Synthetic composite promoters (e.g., promoters integrating NF-κB and STAT3 elements) exploit constitutively active oncogenic pathways of tumors to further restrict OV activity [44]. In addition to regulating viral replication, tumor-specific promoters are also used in vivo to control the expression of therapeutic transgenes (e.g., GM-CSF, IL-12, checkpoint inhibitors). This ensures that immunostimulatory molecules are preferentially expressed within the TME, minimizing systemic toxicity while maximizing local efficacy [45,46]. Thus, tumor-specific promoters represent a powerful molecular biology approach that not only improves OV safety but also enables precision targeting and programmable payload delivery in cancer immunotherapy.

Despite their conceptual appeal, promoter-targeting strategies face multiple biological, technical, and translational limitations that constrain their clinical robustness:

1. **Promoter leakiness and off-target expression:** Tumor-specific promoters often retain residual activity in some normal or regenerating tissues. For instance, the hTERT promoter, although largely silent in quiescent cells, can be transiently activated in normal stem cells, endothelial cells, and inflamed tissues, raising potential safety concerns [47]. Similarly, the survivin promoter may exhibit basal activity in proliferating hematopoietic progenitors or during wound healing. Such leakiness can lead to unintended viral replication or off-target transgene expression, particularly problematic when expressing potent cytokines such as IL-12.
2. **Tumor heterogeneity and dynamic regulation:** Promoter activity is rarely uniform across a tumor mass. Intratumoral variation in telomerase or survivin levels can result in patchy replication and incomplete oncolysis. Moreover, hypoxia-responsive or inflammation-inducible promoters fluctuate with microenvironmental cues, leading to inconsistent viral gene expression. For example, an in vivo study reported that HIF-driven OVs may lose replication potential if tumors reoxygenate following therapy or vascular remodeling, reducing efficacy [48].
3. **Epigenetic silencing and context dependence:** Exogenous promoter sequences introduced into viral genomes can undergo epigenetic suppression through DNA methylation or histone modification, leading to transcriptional shutdown during extended viral replication. This phenomenon has been observed with CMV and hTERT promoters in long-term passaging studies [49]. Moreover, promoter strength is often context-dependent—affected by viral backbone, insertion site, and host transcription factor availability—making preclinical predictability difficult.
4. **Reduced viral fitness and replication kinetics:** Restricting essential viral genes to tumor-specific promoters can impair viral replication rate compared with constitutive expression. For instance, under hypoxic conditions, both in vitro and in vivo models showed that hTERT-driven adenoviruses often replicate more slowly than wild-type or E1A-driven counterparts, potentially limiting spread and cytolytic potency. Strategies combining promoter regulation with compensatory mutations or dual-control systems are under development but remain experimentally complex [50].
5. **Manufacturing and regulatory challenges:** Incorporation of large or synthetic promoters increases viral genome size and genetic instability during production. Maintaining promoter integrity and activity across manufacturing batches is technically demanding.
6. **Payload-specific safety considerations:** Even when tumor-specific promoters restrict transgene expression, cytokine payloads such as IL-12, TNF-α, or IFN-β can still diffuse beyond the tumor, potentially inducing systemic inflammation or vascular leak syndromes. Clinical experience with IL-12-armed OVs underscores the need for tightly regulated or inducible promoter systems to mitigate toxicity [51].

#### 3.1.3. MicroRNA (miRNA) Targeting

A more recent biotechnology strategy to enhance the tumor selectivity and safety of OVs involves engineering viral genomes with miRNA target sequences. By incorporating tandem repeats of binding sites complementary to specific host miRNAs into essential viral genes, viral replication can be selectively suppressed in normal tissues where these miRNAs are expressed, while permitting replication in tumor cells with reduced or absent expression of those miRNAs [52]. For instance, miR-122 is abundantly expressed in hepatocytes. It was demonstrated in vivo using murine infection models that incorporating miR-122 target sites into vesicular stomatitis virus (VSV) or adenoviral backbones suppresses replication in normal liver, reducing hepatotoxicity, while allowing replication in liver tumors that downregulate miR-122 [53]. HSV or measles virus vectors have been engineered with miR-7 target sites to reduce, in vivo, neurotoxicity, since neurons express high miR-7 levels but glioblastomas often do not [54]. As a tumor-suppressor miRNA frequently downregulated in cancers, miR-34a target sequences can be inserted into OV genomes to restrict replication in normal tissues, while maintaining replication in malignant cells. As demonstrated by an in vivo study, and to prevent cardiac toxicity, miR-199a sites have been introduced into adenovirus genomes, reducing replication in cardiomyocytes but sparing tumor cells where this miRNA is suppressed [55].

Despite its elegance and demonstrated preclinical utility, miRNA-based regulation faces several **biological, design, and translational constraints** that limit its current clinical application:

1. **Tumor heterogeneity and dynamic miRNA expression:** The expression of many regulatory miRNAs varies not only between patients but also across tumor regions and during disease progression. For instance, miR-122 or miR-34a levels can fluctuate in response to inflammation, hypoxia, or therapy-induced stress. Consequently, viral replication control based solely on presumed “tumor-absent” miRNAs may be unreliable, risking incomplete viral replication or off-target expression in normal tissues where miRNA levels transiently drop [56].
2. **Incomplete repression and saturation effects:** Even with multiple tandem target sites, miRNA-mediated repression is rarely absolute. Residual low-level expression of essential viral genes may still permit limited replication in protected tissues, particularly at high viral doses. Conversely, excessive insertion of target repeats can destabilize viral transcripts or overwhelm host miRNA machinery, potentially leading to unpredictable off-target effects or miRNA sequestration (“sponging”), which might dysregulate host gene networks [57].
3. **Evolutionary escape and genetic instability:** During serial replication, OVs may undergo mutational loss or deletion of inserted miRNA target sequences, leading to escape variants that replicate indiscriminately. RNA viruses such as VSV or measles, which possess high mutation rates, are particularly prone to such deletions. Maintaining sequence integrity requires careful vector design and stringent manufacturing controls [58,59].
4. **Context dependence and cross-reactivity:** Some miRNAs exhibit overlapping seed sequences or are co-expressed in unexpected tissues, resulting in unanticipated repression in off-target organs. For example, miR-199a and related family members share sequence similarity that can extend viral repression beyond the intended cardiac tissue, potentially diminishing oncolytic potency. This underscores the importance of comprehensive miRNA profiling during vector design.
5. **Translational and regulatory hurdles:** Implementing miRNA-detargeted OVs in clinical trials demands detailed mapping of miRNA distribution in human tissues and across tumor subtypes, which remains incomplete for many cancers. Furthermore, regulators require robust preclinical toxicology data to confirm that miRNA-mediated repression remains stable under inflammatory, hypoxic, or regenerative conditions—contexts that can alter miRNA levels dramatically.

#### 3.1.4. Retargeting

Retargeting is a central molecular biotechnology strategy to enhance the tumor tropism and infectivity of OVs. This involves modifying viral capsid or envelope proteins to redirect viral binding and entry toward tumor-associated receptors, while reducing recognition of receptors commonly expressed on normal tissues. By engineering viral surface proteins, OVs can achieve greater specificity, reduced off-target infection, and enhanced penetration into tumor masses [60]. In adenoviruses, viral tropism is largely determined by the fiber knob domain, which mediates initial attachment via the coxsackie and adenovirus receptor (CAR). Many tumor cells, however, downregulate CAR, leading to poor adenoviral infection. To overcome this, researchers have created in vitro chimeric adenoviruses in which the Ad5 fiber knob is replaced with knobs from other serotypes with different receptor specificities. For example, Ad5/3 chimeras replace the Ad5 knob with that of Ad3, redirecting binding to desmoglein-2 (DSG2) and CD46, both frequently upregulated in tumors [61,62]. On the other hand, Ad5/35 chimeras target CD46 as a primary receptor, improving infection efficiency in hematologic malignancies and gliomas as shown in vivo [63]. Beyond serotype chimeras, tumor selectivity can be achieved by genetically incorporating peptide ligands, single-chain antibodies (scFvs), or growth factor motifs into viral surface proteins. For example: Insertion of RGD motifs into the adenovirus fiber knob or penton base allows integrin-mediated entry, exploiting αvβ3/β5 integrins that are highly expressed in many cancers [64]. Display of EGFR-binding ligands or scFvs against HER2/neu on viral capsids enables selective entry into tumors overexpressing these receptors. For instance, an in vivo study showed that Measles virus can be retargeted by engineering its hemagglutinin (H) protein to bind tumor receptors such as CD20 or EGFR, while ablating native binding to CD46/SLAM [65]. HSV glycoproteins gD or gB can be modified to redirect viral entry toward tumor-enriched receptors, such as HER2. Vesicular stomatitis virus (VSV) glycoprotein G has also been engineered with tumor-specific ligands, improving selective entry [60].

Although receptor retargeting has markedly refined the precision of OVs, multiple biological, immunological, and practical hurdles continue to constrain its clinical translation.

1. **Tumor receptor heterogeneity** remains one of the foremost obstacles. Expression of target molecules such as CD46, EGFR, HER2, ICAM-1, and integrins often varies among tumor regions and evolves under therapy. For example, Ad5/3 adenoviruses efficiently infect DSG2-high ovarian tumors but fail in DSG2-poor lesions, and HER2-retargeted HSVs show uneven replication in breast cancers with heterogeneous HER2 expression. In glioblastoma, subsets of cells lacking CD46 or nectin-1 escape infection even with optimized adenoviral or HSV vectors. This was demonstrated using in vitro models [66].
2. **Expression in normal tissues.** Most tumor-associated receptors are not truly cancer-specific. Low-level expression of HER2 in cardiomyocytes and epithelial cells, or of EGFR in skin and respiratory mucosa, can cause off-target toxicity. EGFR-targeted measles viruses have induced pulmonary inflammation in preclinical models, while CD46-tropic vectors (e.g., Ad5/35) may infect immune or endothelial cells, complicating systemic delivery safety [67].
3. **Receptor accessibility.** From a structural standpoint, modifying viral surface proteins often compromises assembly and infectivity. In adenoviruses, large peptide insertions into the fiber knob can disrupt trimerization and reduce viral yield; RGD-modified Ad5 particles show improved tumor entry but decreased stability. In HSV, engineering gD to recognize HER2 instead of nectin-1 or HVEM can impair fusion efficiency and lower replication rates. Similarly, measles virus H-protein retargeting to CD20 or CEA can destabilize the H–F complex, reducing fusion potency [67].
4. **Immune-mediated neutralization.** Immune neutralization also poses a major translational barrier. Retargeted vectors derived from common serotypes (Ad5, HSV-1, measles) remain susceptible to pre-existing antibodies and complement attack. Ad5/3-D24-GM-CSF and ONCOS-102—though potent in situ—require intratumoral administration because systemic delivery leads to rapid neutralization. Even novel surface ligands can inadvertently expose neo-epitopes, heightening immunogenicity [67].
5. **Dynamic receptor regulation.** Receptor expression is also dynamic and context-dependent. Tumor cells may downregulate or shed receptors after initial viral binding, an antiviral escape observed with integrin- and DSG2-targeted adenoviruses. Cytokine-driven or IFN-mediated responses can alter receptor glycosylation, further diminishing secondary infection cycles [68].
6. **Tumor architecture**. In addition, biophysical barriers within the TME—dense extracellular matrix, stromal fibroblasts, and high interstitial pressure—limit viral diffusion. For instance, an in vivo study showed that RGD-modified adenoviruses and HER2-targeted HSVs typically show peripheral infection with minimal spread into the hypoxic tumor core. Combining retargeting with matrix-degrading enzymes (e.g., hyaluronidase or relaxin) or stromal-modulating agents is being explored to overcome this limitation [69].

### 3.2. Payload Incorporation

Arming OVs with therapeutic payloads is a powerful biotechnology strategy to augment their anti-tumor effects.

#### 3.2.1. Cytokines/Chemokines

One of the most successful payload strategies has been the incorporation of immune-stimulatory cytokines. The prototype is Talimogene laherparepvec (T-VEC), an HSV-1-based OV engineered to express Granulocyte–Macrophage Colony-Stimulating Factor (GM-CSF). GM-CSF recruits and matures DCs within the TME, enhancing the uptake and cross-presentation of TAAs. This facilitates priming of CD8^+^ T cells and broadens systemic antitumor immunity [70]. IL-12 is a heterodimeric cytokine (p35 + p40 subunits) that induces STAT4 activation and IFN-γ production from T and NK cells. In OVs, IL-12 expression enhances Th1 polarization, increases cytotoxicity of CD8^+^ T cells, and upregulates MHC class I expression on tumor cells, thereby promoting antigen visibility [71]. IL-15 supports homeostatic proliferation and survival of NK cells and memory CD8^+^ T cells. OV-mediated delivery of IL-15 induces phosphorylation of JAK1/3–STAT5, leading to enhanced NK cytotoxicity and durable T cell responses, in vivo, particularly in poorly immunogenic tumors [72]. IL-18 synergizes with IL-12 to further augment IFN-γ secretion. It activates MyD88/NF-κB signaling in immune cells, driving NK and CD8^+^ T cell recruitment and promoting anti-tumor cytotoxicity [73]. A member of the IL-1 family, IL-36γ activates NF-κB and MAPK signaling via the IL-36 receptor on DCs, macrophages, and epithelial cells. OVs expressing IL-36γ stimulate strong DC maturation and proinflammatory cytokine cascades, reshaping the TME from immunosuppressive to immunogenic [74]. OVs engineered to express IFN-α or IFN-β enhance antiviral and antitumor immunity by upregulating ISGs (e.g., PKR, OAS, MxA), enhancing antigen presentation via MHC-I, and promoting apoptosis in tumor cells. IFN-γ, expressed from OVs, further amplifies CD8^+^ T cell cytotoxicity and inhibits angiogenesis [75]. As a TNF superfamily member, LIGHT binds HVEM (TNFRSF14) and LTβR, activating NF-κB signaling and enhancing T cell co-stimulation. OV-mediated LIGHT expression leads to increased T cell infiltration, enhanced DC function, and tumor vasculature normalization [76]. Chemokines can also be incorporated to recruit effector cells into the TME. For example, an in vitro study reported that OV-encoded CCL5 (RANTES) engages CCR5 receptors on NK cells, CD8^+^ T cells, and DCs, driving their migration into tumors. This not only increases immune cell density but also enhances effector-to-target ratios, improving tumor clearance [76]. Together, cytokine- and chemokine-armed OVs act as in situ cancer vaccines, reshaping the TME and bridging innate and adaptive immunity. However, despite this compelling rationale, cytokine/chemokine arming comes with important limitations, safety considerations, and translational bottlenecks that must be addressed for durable clinical benefit.

*1. Systemic spillover and dose control.* Even when transgenes are placed under tumor-active promoters, payloads can diffuse beyond the lesion via lymphatics/vasculature, causing cytokine-related toxicities (fever, hypotension, hepatotoxicity). This is acute for IL-12 (capillary leak, liver enzyme rise) and type I IFNs (flu-like syndromes, cytopenias). Intratumoral dosing does not fully prevent leakage, and repeated injections can accumulate systemic levels [77].

*2. Pleiotropy and context-dependence*. Cytokines are non-linear actuators: the same molecule can help or hurt depending on timing, cell type, and local milieu. GM-CSF can mature DCs but also expand MDSCs and skew macrophages to suppressive phenotypes, blunting T-cell priming. CCL5 (RANTES) recruits CD8^+^/NK cells, but in some tumors also draws Tregs and CCR5^+^ M2 macrophages, increasing immunosuppression. IFN-α/β boosts antigen presentation but also induces PD-L1, IDO, and apoptosis of activated T cells, potentially tightening feedback brakes [78].

*3. Antiviral overshadowing of oncolysis*. Type I IFNs produced by the payload (or secondarily induced by IL-12/IL-18) upregulate ISGs (PKR/OAS/Mx) in infected tumor cells, which can abort viral replication and curtail intratumoral spread—great for safety, bad for potency. This “self-sabotage” is especially visible with IFN-expressing HSV/Ad vectors and in tumors with intact JAK/STAT signaling [79].

*4. Target-side resistance and pathway loss*. Many advanced tumors harbor IFN pathway defects (JAK1/2, STAT1 loss) or antigen presentation defects (β2M, HLA class I). IL-12/IFN payloads presuppose intact sensing and MHC-I upregulation; when absent, you get payload-insensitive tumors despite robust cytokine expression. Conversely, stromal or endothelial cells with intact IFN responses may bear the brunt of toxicity [80].

*5. Gradient biology and trafficking paradoxes*. Chemokines must form stable intratumoral gradients to pull effectors in. High bulk expression can “flatten” gradients or trap lymphocytes in perivascular cuffs. CC-chemokines (e.g., CCL5) may recruit mixed leukocyte pools; CXCR3-biased axes (CXCL9/10/11) better enrich for Th1/CD8^+^ but are IFN-dependent. Dense ECM and hypoxia further decouple chemokine expression from effective infiltration [81].

*6. Receptor desensitization and exhaustion*. Sustained high chemokine/cytokine levels cause GPCR desensitization (β-arrestin) and T-cell exhaustion (PD-1, TIM-3 upregulation). IL-15 overdrive can trigger NK hyperactivation → attrition, while chronic IL-12 drives terminal differentiation at the expense of memory [82].

*7. Payload format matters. Soluble payloads diffuse*. membrane-tethered or locally anchored versions reduce spillover but may limit bystander reach. IL-15/IL-15Rα sushi complexes improve potency yet raise durability-linked safety flags. TNF-superfamily members (e.g., LIGHT) normalize vasculature in some contexts, but can inflame endothelium and increase thrombosis risk in others [83].

*8. Genetic stability and manufacturing*. Large inserts (multi-cytokine cassettes, polycistrons) increase genome instability, lower titers, and complicate QC (payload copy number, expression heterogeneity). For RNA OVs (e.g., VSV, measles), high mutation rates risk payload loss; for large DNA OVs (HSV, VV), cargo size and recombination hotspots reduce yields and batch consistency [84].

*9. Clinical signal variability*. While T-VEC (GM-CSF) validated the concept, its systemic efficacy is modest; combinations (e.g., anti-PD-1) have yielded mixed late-phase results. Many IL-12/IFN-armed OVs show impressive pharmacodynamics but limited objective responses outside accessible, inflamed lesions—highlighting delivery and resistance issues above [85].

#### 3.2.2. Immune Checkpoint Modulators

Another important payload strategy is engineering OVs to deliver immune checkpoint inhibitors (ICIs) directly within the TME. By locally expressing antibodies, minibodies, or soluble receptor decoys against inhibitory receptors, OVs can reinvigorate exhausted T cells while minimizing systemic immune-related adverse events. Immune checkpoints such as PD-1/PD-L1 and CTLA-4 suppress T cell activity by dampening TCR signaling cascades. PD-1 engagement recruits SHP-2 phosphatase, which dephosphorylates CD3ζ and ZAP-70, attenuating downstream PI3K/AKT and MAPK signaling, thereby reducing IL-2 production and effector function [86]. CTLA-4 competes with CD28 for binding to B7 ligands (CD80/CD86) on APCs, preventing costimulatory signaling and leading to T cell anergy. Blocking these pathways restores NFAT/NF-κB activation, cytokine production, and cytotoxic granule release, ultimately enhancing tumor clearance [87]. Newcastle Disease Virus (NDV) has been engineered to express anti-PD-1 scFvs, enhancing CD8^+^ T cell infiltration and granzyme B release within the TME. This reverses T cell exhaustion while avoiding systemic antibody exposure [87]. Recombinant Vaccinia Virus (VV) has been armed with anti-CTLA-4 or anti-PD-L1 minibodies, which block inhibitory signaling in situ, leading to increased intratumoral IFN-γ^+^ CD8^+^ T cells and decreased Treg activity in vivo. Adenoviruses encoding soluble PD-1 decoys or VSV encoding anti-PD-L1 minibodies have also shown tumor-selective checkpoint blockade, potentiating immune memory and synergy with systemic ICIs [87].

Although engineering OVs to express immune checkpoint inhibitors (ICIs) locally within the TME represents a promising approach to augment antitumor immunity while reducing systemic toxicity, several biological and translational limitations still constrain its therapeutic impact.

(1) Redundancy of immunosuppressive pathways in the TME. Tumor immune suppression is multifactorial. While PD-1/PD-L1 and CTLA-4 blockade restores some T cell activity, other inhibitory axes such as TIM-3, LAG-3, TIGIT, and VISTA, along with the adenosine and IDO metabolic pathways, often compensate, sustaining T cell dysfunction and immune evasion. This redundancy was illustrated in preclinical models of melanoma and pancreatic cancer, where PD-L1 blockade alone failed to overcome the suppressive activity of myeloid-derived suppressor cells (MDSCs) and tumor-associated macrophages (TAMs). Therefore, single-checkpoint payloads in OVs may have transient or incomplete effects unless combined with myeloid reprogrammers (e.g., IL-12, CD40L, or STING agonists) [86].

(2) Epigenetic fixation of T-cell exhaustion. Another barrier arises from the epigenetic fixation of T cell exhaustion. Once chronic antigen exposure and inhibitory signaling establish an exhausted phenotype, chromatin accessibility in effector loci such as IFNG, GZMB, and IL2 becomes permanently repressed. This means that PD-1 or CTLA-4 blockade delivered by OVs cannot fully rejuvenate terminally exhausted T cells. Meanwhile, selective pressure from OV-induced inflammation can drive antigen-loss variants or MHC-I downregulation via B2M or JAK1/2 mutations, as documented, in vivo, in colorectal carcinoma models treated with adenovirus-encoded PD-1 decoys [88]. The antiviral immune response further limits this approach. Type I and III IFNs, although essential for antitumor priming, simultaneously suppress viral replication and transgene expression. For example, vesicular stomatitis virus (VSV) engineered to express anti-PD-L1 minibodies demonstrated robust early transgene expression that rapidly declined once the host produced neutralizing antibodies, curtailing its therapeutic window. Likewise, patients previously exposed to vaccinia-based vaccines may harbor high titers of neutralizing antibodies, restricting viral spread upon repeated administration.

(3) Genetic instability of large therapeutic inserts. At the molecular level, efficient and safe antibody expression within infected tumor cells poses additional hurdles. Many OVs cannot accommodate full-length IgG constructs because of genome size constraints; therefore, smaller antibody fragments such as single-chain variable fragments (scFvs) or minibodies are typically used. However, these smaller molecules exhibit shorter half-lives and altered effector functions due to the absence of Fc domains. Moreover, viral replication factories often lack the proper post-translational machinery for complex glycosylation, leading to misfolded or less stable antibodies. For example, HSV-1 expressing anti-CTLA-4 scFvs produced functional but partially degraded fragments within 48 h post-infection. The choice between secreted versus membrane-tethered formats also affects local retention: while secreted minibodies diffuse widely, tethered scFvs require direct cell–cell contact to exert function but risk proteolytic shedding within the TME [89]. The genetic stability of large therapeutic inserts is another concern. During serial replication, non-essential transgene cassettes can be deleted or silenced. Studies with VV-anti-PD-L1 constructs showed loss of transgene expression after multiple viral replication cycles, attributed to selective pressure against large genome inserts. This instability complicates consistent dosing and may reduce efficacy over repeated treatments [90].

(4) Pharmacokinetic, pharmacodynamic, and manufacturing challenges. From a translational perspective, pharmacokinetic and pharmacodynamic assessment is extremely challenging. Viral titers and minibody concentrations vary widely across tumors, and there is no straightforward biomarker to measure intratumoral antibody levels in real time. In NDV encoding anti-PD-1 scFvs, for instance, local concentrations peaked within 48 h but were undetectable systemically, leaving uncertainty about dose–response relationships. Excessive viral dosing risks overt local inflammation or necrosis, while insufficient infection yields subtherapeutic exposure. Manufacturing such combination biologics introduces further complexity. Each preparation must ensure consistent infectious titer, genetic integrity, and functional transgene expression. Quality control assays must confirm both viral potency and immune checkpoint blockade activity. Regulatory evaluation becomes difficult because these agents combine a replicating viral component with a therapeutic antibody, blurring the classification between gene therapy and biologic drug [91].

(5) Clinical delivery constraints and localized toxicity. Finally, clinical logistics and safety concerns remain significant. Intratumoral injection restricts the approach to accessible lesions such as cutaneous melanoma or superficial head and neck tumors, whereas deep-seated or metastatic cancers require image-guided delivery. Moreover, local checkpoint release can still induce focal immune-related adverse events. Cases of localized colitis-like inflammation have been observed when adenovirus-encoded anti-CTLA-4 was administered intrarectally, and cutaneous necrosis was reported following vaccinia-mediated PD-L1 blockade in melanoma lesions, underscoring that local therapy does not eliminate toxicity but rather redistributes it [92].

#### 3.2.3. BiTEs (Bispecific T-Cell Engagers)

BiTEs (bispecific T-cell engagers) are synthetic antibody fragments that combine two single-chain variable fragments (scFvs): one specific for CD3ε on T cells and the other for a TAA. By bridging T cells and tumor cells, BiTEs force the formation of an artificial immunological synapse, resulting in TCR-independent T cell activation [93]. This leads to phosphorylation of CD3ζ chains, activation of Lck/ZAP-70 kinases, downstream Ca^2+^ influx, and signaling through NFAT/NF-κB, culminating in perforin/granzyme release and tumor cell apoptosis. Engineering OVs to express BiTEs provides local and sustained BiTE production within the TME, overcoming the short serum half-life and systemic toxicity of intravenously administered BiTEs [94]. An adenovirus armed with a MUC16-BiTE was shown to redirect polyclonal T cells against ovarian cancer cells, enhancing infiltration, IFN-γ secretion, and reversing the immunosuppressive TME. Other OVs, including adenovirus and vaccinia backbones, have been engineered to secrete BiTEs targeting EGFR or EpCAM, leading to enhanced CD8^+^ T cell activity and robust tumor killing, even in tumors with low MHC-I expression. Next-generation OVs are being designed to encode dual BiTEs or TriTEs, simultaneously engaging T cells and NK cells to broaden the immune attack and address tumor heterogeneity [95].

While arming OVs with bispecific T-cell engagers (BiTEs) represents an innovative strategy to locally redirect cytotoxic lymphocytes against tumor cells, several biological, immunological, and translational limitations constrain its clinical utility [96].

(1) Risk of cytokine release syndrome and systemic immune activation. The potent, antigen-independent activation of T cells mediated by BiTEs carries a risk of cytokine-release syndrome (CRS) even when expression is restricted to TME. Locally produced BiTEs can diffuse beyond the infection site, especially in highly vascularized or necrotic tumors, resulting in systemic spillover of IL-6, TNF-α, and IFN-γ. In preclinical models using adenovirus-encoded CD3 × EpCAM BiTEs, elevated cytokine levels and transient weight loss were observed despite the intended confinement of expression [97]. While oncolytic viruses are generally better tolerated than many systemic immunotherapies, CRS and irAEs remain clinically relevant concerns, particularly in combination regimens involving immune checkpoint inhibitors, cytokine-armed viruses, or BiTE-expressing platforms. These toxicities reflect the intended immune activation but may influence dose intensity, scheduling, and patient eligibility. Importantly, available clinical data indicate that most OV-associated CRS cases and irAEs are low to moderate in severity and clinically manageable using established interventions such as corticosteroids, IL-6 blockade, or temporary treatment interruption. However, the risk increases in settings involving potent immunostimulatory payloads or systemic viral delivery, underscoring that toxicity represents a context-dependent translational constraint rather than a prohibitive barrier. Accordingly, multiple mitigation strategies are being pursued, including localized intratumoral delivery, tumor-restricted or inducible transgene expression, attenuated viral backbones, and careful treatment sequencing to align OV administration with immune recovery, supporting continued clinical development with appropriate safety oversight [97].
(2) Antigen heterogeneity and immune escape. Another important limitation arises from antigen heterogeneity and immune escape. Many solid tumors exhibit spatially and temporally variable expression of TAAs such as MUC16, EpCAM, or EGFR. OVs encoding single-target BiTEs may effectively eliminate antigen-positive subclones but leave antigen-negative variants intact, promoting rapid selection for escape mutants [98].
(3) Limited control over BiTE expression levels and stoichiometry. From a mechanistic perspective, BiTE expression levels and stoichiometry within the TME are difficult to control. Excessive local BiTE concentrations may trigger T cell “over-activation” and local tissue necrosis, while insufficient expression fails to achieve durable synapse formation or sustained cytotoxicity. Moreover, the short half-life and rapid turnover of BiTEs in protease-rich, hypoxic tumor microenvironments can further reduce their bioavailability.
(4) Dependence on viral replication kinetics and antiviral immunity. OVs that replicate quickly, such as vaccinia or VSV, may produce a burst of BiTEs but are rapidly cleared by antiviral immunity, limiting the duration of immune engagement.
(5) Tumor microenvironment-imposed barriers to immune engagement. The immunological contexture of the TME also dictates BiTE efficacy. Dense fibrotic stroma, abnormal vasculature, and myeloid-derived suppressor cells can physically and metabolically hinder T cell infiltration and activation. In “cold” tumors characterized by low T cell density or defective antigen presentation, BiTEs may have little substrate for engagement [99].
(6) Manufacturing, regulatory, and quality control challenges. Translationally, the manufacturing and regulatory landscape for OV-encoded BiTEs remains complex. These are dual-entity biologics whose potency depends on both viral replication and functional bispecific expression. Batch-to-batch consistency must be verified for infectious titer, BiTE concentration, binding affinity, and in vitro cytotoxicity—parameters not yet standardized for gene-encoded antibody fragments [100].
(7) Residual safety concerns despite localized expression. Finally, safety concerns persist despite localized expression. Viral shedding or leakage of BiTE proteins into systemic circulation can lead to unintended immune activation or off-target effects. In particular, hepatotoxicity and pulmonary inflammation have been reported in murine models where BiTEs targeted antigens with low basal expression in normal tissues [101].

#### 3.2.4. Other Therapeutics

Beyond cytokines, chemokines, and checkpoint inhibitors, OVs can be armed with suicide genes that mediate localized conversion of systemically administered prodrugs into cytotoxic metabolites within the tumor microenvironment. This suicide gene–prodrug system amplifies tumor cell killing, enhances bystander effects, and minimizes systemic toxicity. For instance, HSV thymidine kinase (HSV-tk) phosphorylates nucleoside analogs such as ganciclovir (GCV) into monophosphate derivatives, which are further phosphorylated by cellular kinases into toxic triphosphates. These metabolites incorporate into DNA, causing chain termination and apoptosis. Importantly, toxic metabolites diffuse through gap junctions into neighboring uninfected cells, creating a bystander effect that expands tumor killing beyond directly infected cells. Bacterial or yeast Cytosine deaminase (CD) converts the non-toxic prodrug 5-fluorocytosine (5-FC) into 5-fluorouracil (5-FU), a potent chemotherapeutic. 5-FU is further metabolized to FdUMP and FUTP, inhibiting thymidylate synthase and incorporating into RNA/DNA, resulting in impaired DNA synthesis, RNA processing defects, and tumor cell apoptosis. In vitro human tumor cell models demonstrated that recombinant vaccinia virus encoding CD or HSV-tk has been shown to selectively deliver suicide genes to colorectal tumors, producing high intratumoral drug concentrations while sparing healthy tissues [102].

### 3.3. Combination Therapies

**Figure 2:**
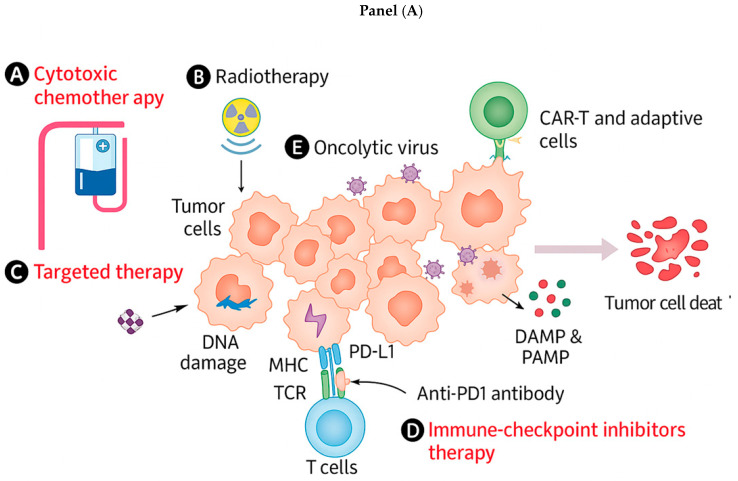

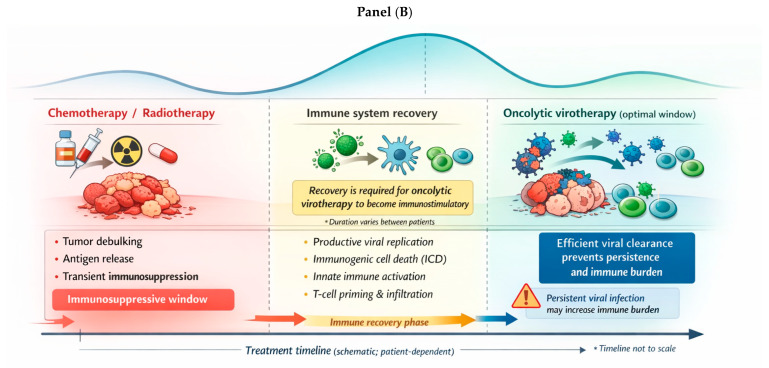
**Integrated therapeutic strategies enhancing tumor cell killing and antitumor immunity.** Panel (**A**) schematic illustrates how multiple cancer treatment modalities converge to induce tumor cell death and strengthen immune responses. A: Cytotoxic chemotherapy kills tumor cells directly and promotes ICD. B: Radiotherapy generates DNA damage, increasing tumor antigen release and sensitizing cells to immune clearance. C: Targeted therapy disrupts oncogenic signaling pathways, contributing to tumor stress and improved antigen presentation. D: Immune-checkpoint inhibitors (anti-PD-1) block PD-1/PD-L1 interactions, reactivating exhausted T cells and enhancing their cytotoxic function. E: OVs selectively infect and lyse tumor cells, releasing DAMPs and PAMPs that recruit and activate immune cells, while synergizing with CAR-T and other adoptive cell therapies. Together, these complementary therapeutic approaches amplify immune activation and collectively drive robust tumor cell destruction. Panel (**B**) Timing-dependent integration of conventional therapies and oncolytic virotherapy. Chemotherapy and radiotherapy induce tumor debulking and antigen release but are associated with transient immunosuppression. A recovery phase is required to restore immune competence before oncolytic virus administration can effectively stimulate antitumor immunity. When delivered during an immune-recovered window, oncolytic virotherapy promotes immunogenic cell death, innate immune activation, and T-cell priming. Efficient viral clearance is essential to prevent persistent infection and excessive immune burden. The duration of immune suppression and recovery varies between patients; therefore, the timeline is schematic and not drawn to scale.

Combining OVs with other modalities can lead to synergistic effects (Figure 2):

#### 3.3.1. Immune Checkpoint Inhibitors (ICIs)

The immune activation induced by OVs can be significantly amplified by combining them with immune checkpoint inhibitors (ICIs). OVs initiate antitumor immunity by causing ICD and releasing TAAs, DAMPs, and viral pathogen-associated molecular patterns (PAMPs). These events lead to DC maturation, cross-presentation of antigens, and priming of tumor-specific CD8^+^ T cells. However, many of these T cells become functionally exhausted due to checkpoint engagement within the tumor microenvironment (TME). Engagement of PD-1 on T cells by PD-L1 on tumor or myeloid cells recruits SHP-2 phosphatase, dephosphorylating CD3ζ and ZAP-70, and inhibiting downstream PI3K/AKT and MAPK signaling. This leads to decreased cytokine production, impaired proliferation, and T cell exhaustion. CTLA-4 competes with CD28 for binding to B7 ligands (CD80/CD86) on antigen-presenting cells (APCs). By outcompeting CD28, CTLA-4 prevents costimulatory signaling, resulting in poor IL-2 production and impaired clonal expansion of T cells. By combining OVs with ICIs, two complementary mechanisms are harnessed: (1) OVs prime immunity—by releasing TAAs and reshaping the TME into an inflamed, immunogenic niche and (2) ICIs sustain immunity—by removing inhibitory signals (PD-1/PD-L1, CTLA-4), preventing exhaustion and allowing expanded effector and memory T cell responses. The most advanced clinical example is T-VEC (HSV-1 expressing GM-CSF) combined with pembrolizumab (anti-PD-1), which showed durable responses in advanced melanoma patients. Preclinical models combining OVs with anti-PD-1 or anti-PD-L1 antibodies demonstrate enhanced CD8^+^ T cell infiltration, IFN-γ secretion, and tumor regression compared to monotherapy. Dual-checkpoint blockade strategies, such as combining CTLA-4 and PD-L1 inhibition with OV therapy, are being investigated to further augment T cell function while broadening the range of rescued exhausted T cells. Instead of systemic antibody delivery, OVs are also being engineered to express checkpoint inhibitors locally (e.g., anti-PD-L1 scFvs, anti-CTLA-4 minibodies), concentrating blockade within the TME and minimizing systemic immune-related adverse events. Thus, OV–ICI combinations represent a synergistic paradigm: OVs act as “tumor vaccines” that expose the immune system to antigens, while ICIs act as “immune brakes release,” unleashing sustained and effective antitumor immunity [103].

Despite encouraging preclinical and early clinical outcomes, the therapeutic promise of OV–ICI combinations remains highly heterogeneous, tumor-dependent, and context-specific. While small-scale studies have demonstrated objective responses and durable benefit in selected patients, larger randomized trials have not yet consistently validated this synergy. For instance, the phase Ib MASTERKEY-265 trial of Talimogene laherparepvec (T-VEC; HSV-1/GM-CSF) plus pembrolizumab showed increased intratumoral CD8^+^ T-cell infiltration and durable response rates of approximately 40% in advanced melanoma, suggesting a favorable immune activation profile. However, in the subsequent phase III MASTERKEY-265/KEYNOTE-034 trial, the combination failed to significantly improve progression-free survival (PFS) or overall survival (OS) compared to pembrolizumab alone, indicating that early immunologic responses do not always translate into long-term clinical benefit [104].

Similarly, Pexa-Vec (JX-594; vaccinia virus expressing GM-CSF and β-galactosidase) combined with nivolumab or ipilimumab produced mixed results across hepatocellular carcinoma (HCC) and renal cell carcinoma (RCC) trials. While some patients exhibited partial responses and increased infiltration of IFN-γ–producing CD8^+^ T cells, others demonstrated minimal benefit due to rapid antiviral neutralization and poor intratumoral viral propagation. In the PHOCUS trial (NCT02562755), Pexa-Vec monotherapy also failed to extend survival in advanced HCC, highlighting the challenge of systemic delivery and immune clearance in immunologically “cold” or fibrotic tumors. Likewise, combinations involving Coxsackievirus A21 (CVA21; Cavatak) with anti-PD-1 therapy in advanced melanoma or bladder cancer produced promising early immune activation signatures (increased PD-L1 expression and T-cell infiltration), but failed to yield consistent durable responses, likely reflecting pre-existing antiviral immunity, TME hypoxia, and variable viral tropism across tumor histotypes [105].

Mechanistically, the variability in viral replication efficiency, IFN signaling, and baseline immune contexture represents a key determinant of therapeutic outcome. Tumors with high intrinsic IFN-I responses may rapidly suppress OV replication, curtailing oncolysis and transgene expression before effective antigen release or immune priming occurs. Conversely, IFN-defective tumors, such as those harboring STING or JAK/STAT pathway mutations, may support more robust viral propagation but paradoxically show poor responsiveness to ICIs due to impaired antigen presentation and low T-cell recruitment. Thus, the same molecular defect that enhances viral replication can simultaneously blunt adaptive immunity, complicating the design of universally effective OV–ICI regimens [106].

Pre-existing or rapidly induced antiviral immunity further restricts efficacy. Neutralizing antibodies against vaccinia, HSV-1, or adenoviral backbones can prevent secondary infections, reducing the viral load in tumor lesions after repeated dosing. Even within injected tumors, heterogeneous viral distribution—due to necrosis, stromal density, or poor perfusion—creates spatially uneven immune activation, leaving pockets of uninfected tumor cells that can drive relapse [107].

From a clinical safety perspective, immune-related adverse events (irAEs) and excessive inflammation remain important concerns. Although OVs aim to focus immune stimulation locally, systemic immune activation can still occur when viral PAMPs and checkpoint blockade converge, amplifying cytokine cascades. Reported toxicities include localized colitis, dermatitis, cytokine-release-like syndromes, and exacerbation of pre-existing autoimmune disorders. For example, in early Pexa-Vec and T-VEC combination trials, transient flu-like symptoms, fever, and injection-site inflammation were common, reflecting a heightened inflammatory milieu. While generally manageable, such effects raise caution when combining potent immune stimulators with systemic ICIs, particularly in heavily pre-treated or immunologically fragile patients. Another underappreciated challenge lies in tumor heterogeneity and immune escape. OV–ICI therapy predominantly benefits tumors already “warm” or partially inflamed; in immunologically “cold” or myeloid-dominant tumors, such as pancreatic or prostate cancer, the TME often remains resistant despite oncolytic infection. Myeloid-derived suppressor cells (MDSCs), regulatory T cells (Tregs), and TAMs can rapidly reestablish immunosuppression after initial immune activation. Moreover, persistent exposure to viral antigens and checkpoint blockade can drive adaptive resistance, including PD-L1 upregulation, recruitment of Tregs, or antigen loss variants [108].

Therefore, while OV–ICI combinations represent an elegant mechanistic convergence of viral immunogenicity and checkpoint blockade, their clinical maturity remains limited, and the translational landscape is still evolving. To realize their full potential, future development must focus on next-generation viral vectors with improved tumor selectivity, rational sequencing of therapy (e.g., priming with OVs before ICI introduction), and biomarker-driven patient stratification based on IFN signatures, viral receptor expression, and baseline T-cell infiltration. Additionally, engineering OVs to co-deliver immunostimulatory payloads (e.g., IL-12, CD40L, or STING agonists) or localized checkpoint fragments (anti-PD-L1 scFvs) may help overcome systemic toxicity while enhancing local immune engagement [109].

#### 3.3.2. Adoptive Cell Therapies (ACTs)

OVs can act as biological adjuvants that remodel the tumor microenvironment (TME), thereby enhancing the efficacy of adoptive cell therapies (ACTs) such as tumor-infiltrating lymphocytes (TILs), engineered T cell receptor (TCR) therapies, and chimeric antigen receptor (CAR)-T cell therapies. The synergy arises from OV-mediated immune activation combined with the targeted cytotoxicity of ACTs. OVs engineered to express cytokines such as IL-7 and IL-15 provide essential survival and proliferation signals for transferred CAR-T cells. IL-7 enhances T cell homeostatic proliferation via STAT5 activation, promoting long-term persistence. IL-15 augments JAK1/3–STAT5 signaling, expanding NK cells and memory CD8^+^ T cells, improving CAR-T cytotoxicity and durability. OVs can induce ICD that releases TAAs, DAMPs (ATP, HMGB1, calreticulin), and pro-inflammatory cytokines, which reduce local immunosuppression and enhance CAR-T infiltration. OV infection upregulates chemokines such as CXCL9 and CXCL10 within the TME, which attract CXCR3^+^ CAR-T cells, improving their homing and tumor infiltration. OVs expressing bispecific T cell engagers (BiTEs) redirect polyclonal T cells, including adoptively transferred ones, to kill tumor cells even in the absence of MHC-I or uniform antigen expression. This broadens ACT efficacy in antigen-heterogeneous tumors. By encoding local checkpoint inhibitors (anti-PD-1/PD-L1, anti-CTLA-4 minibodies), OVs reduce exhaustion of ACT-expanded T cells and improve effector function [110]. Preclinical studies show that adenoviruses expressing IL-15 or IL-12 enhance CAR-T persistence and expansion within solid tumors. A triple-combination strategy, where OVs both lyse tumor cells and secrete BiTEs, can redirect endogenous T cells and synergize with CAR-T cells to overcome antigen loss. OV-mediated release of TAAs and inflammatory cytokines enhances TIL recruitment, survival, and recognition of antigen-diverse tumor cells [111].

While the rationale for combining OVs with adoptive cell therapies (ACTs) such as TILs, TCR-engineered T cells, and CAR-T cells is compelling, clinical translation has revealed considerable biological, logistical, and safety challenges. Preclinical data suggest strong synergism, yet consistent clinical efficacy in solid tumors remains elusive. The major mechanistic appeal lies in OVs’ ability to reprogram the “cold” tumor microenvironment (TME) into an inflamed niche rich in cytokines and chemokines, thereby supporting T-cell infiltration and survival. However, the same antiviral immune mechanisms that facilitate inflammation can also counterproductively limit viral replication and persistence, curtailing the duration of cytokine or chemokine expression that supports transferred T cells [112].

A central limitation arises from poor trafficking and retention of ACTs within solid tumors, even after OV infection. Although OVs can upregulate chemokines such as CXCL9/CXCL10 to attract CXCR3^+^ CAR-T or TIL populations, the physical and metabolic barriers of the TME—such as dense extracellular matrix, hypoxia, and suppressive myeloid infiltration—still impede T-cell penetration. Moreover, repeated OV administration can lead to fibrotic encapsulation of the injection site, paradoxically hindering later immune cell infiltration [113].

Another challenge is antigen heterogeneity and immune escape. While OVs may broaden antigen exposure through ICD and the release of TAAs, CAR-T cells and TCR-engineered T cells remain restricted to defined targets. As a result, selective pressure from ACTs can drive antigen-negative relapse, as seen in preclinical models combining CAR-T cells with adenoviruses encoding IL-15 or BiTEs, where tumors eventually outgrew due to loss of the targeted antigen. Dual-target or “armored” CARs (e.g., PD-1-dominant negative CARs) and OV-delivered BiTEs or TriTEs partially mitigate this limitation but at the cost of increased genomic complexity and manufacturing difficulty [113].

*Interference between antiviral and antitumor immunity is another recurring issue.* OV infection activates type I IFNs and innate immune responses that, while essential for DC activation and antigen presentation, can also induce a cytokine milieu that suppresses T-cell proliferation or accelerates exhaustion of adoptively transferred cells. For instance, in murine models combining VSV or HSV-1 vectors with CAR-T therapy, excessive type I IFN signaling promoted PD-L1 upregulation on tumor and stromal cells, leading to CAR-T hypofunction. This underscores the need for precise timing and dosing—using OVs as priming agents before, rather than concurrently with, ACT infusion—to avoid mutual interference [113].

*From a translational standpoint, pre-existing antiviral immunity poses a major barrier.* Neutralizing antibodies against adenovirus or vaccinia can rapidly eliminate OVs, reducing transgene expression and shortening the immunostimulatory window that supports CAR-T persistence. In clinical settings, patients who received multiple OV doses before ACT infusion often showed reduced viral titers and lower cytokine induction, resulting in weaker T-cell engraftment. Strategies such as regional administration (e.g., intraperitoneal or hepatic artery infusion), use of nonhuman viral backbones (e.g., VSV, NDV, Maraba virus), or transient immunosuppression may alleviate this issue, but safety and reproducibility remain under study [114].

*Safety concerns are also nontrivial.* Combining OVs and ACTs amplifies immune activation and cytokine production, heightening the risk of cytokine release syndrome (CRS) and immune-related tissue damage. For example, preclinical adenoviral IL-15–CAR-T combinations led to high intratumoral IL-15 concentrations associated with systemic IL-6 elevation and transient weight loss in murine models. Furthermore, unrestrained OV replication in immunocompromised hosts or in the setting of intensive lymphodepletion prior to ACTs could theoretically cause uncontrolled viral dissemination. Careful vector selection and dose titration are thus critical for safety [115].

*Clinical translation remains in early stages.* To date, only a limited number of first-in-human trials have evaluated OV–ACT combinations. A pilot study combining T-VEC with adoptively transferred TILs in advanced melanoma reported increased intratumoral T-cell density and cytokine production, but without significant improvement in overall response rates compared to historical ACT data. Similarly, an early-phase I study using oncolytic adenovirus expressing IL-12 with HER2-specific CAR-T cells in glioblastoma demonstrated feasibility and transient radiographic tumor shrinkage but also significant local edema, underscoring the inflammatory risk. Trials involving DNX-2401 (adenovirus) with anti-EGFRvIII CAR-T cells for glioblastoma and MG1-MARABA virus with NY-ESO-1 TCR-T cells for sarcoma and melanoma are ongoing, but published efficacy data remain preliminary and mixed [116]. Mechanistic limitations are also emerging. Continuous antigen exposure in the inflamed OV-treated TME can accelerate CAR-T exhaustion, characterized by PD-1, TIM-3, and LAG-3 upregulation, and transcriptional reprogramming toward a dysfunctional state. Likewise, the metabolic stress induced by viral infection—hypoxia, lactate accumulation, and nutrient depletion—further compromises ACT metabolism and cytotoxic function. Thus, while OVs can transiently enhance T-cell activation, sustained coexistence within a hostile metabolic and inflammatory milieu may ultimately reduce CAR-T fitness [117].

#### 3.3.3. Targeted Therapies, Epigenetic Therapies and Chromatin Reprogramming Strategies

Combining OVs with targeted therapies is a rapidly evolving approach aimed at exploiting vulnerabilities in tumor signaling and epigenetic regulation. These combinations enhance viral replication, promote ICD, and increase tumor susceptibility to immune clearance. Many tumors harbor mutations in the MAPK pathway, such as BRAF^V600E in melanoma. BRAF inhibitors (vemurafenib, dabrafenib) and MEK inhibitors (trametinib) suppress tumor cell proliferation but often induce compensatory immunosuppressive mechanisms [118]. Preclinical studies demonstrate that BRAF inhibition enhances OV replication by downregulating antiviral IFN-stimulated gene (ISG) expression, thereby rendering tumor cells more permissive to viral infection.

Mechanistically, both in vitro and in vivo studies revealed that OVs in combination with BRAF/MEK blockade lead to: (1) Reduced ERK signaling, which lowers antiviral defenses, (2) Upregulation of melanoma antigens (e.g., MART-1, gp100), boosting T cell recognition and (3) increased T cell infiltration due to chemokine release (CXCL9/10). On the other hand, Histone deacetylase inhibitors (HDACis) such as vorinostat and panobinostat increase chromatin accessibility, promoting transcription of silent viral genes and enhancing OV replication [119]. HDAC inhibition also induces endogenous retrovirus (ERV) expression, leading to dsRNA accumulation, PRR activation (RIG-I, MDA5, TLR3), and type I IFN signaling, thereby augmenting antitumor immunity [120].

Moreover, DNA methyltransferase inhibitors (DNMTis) like decitabine and azacytidine demethylate tumor suppressor promoters and viral entry receptors, enhancing both tumor antigen presentation (via MHC-I upregulation) and OV infectivity [121]. Epigenetic drugs also sensitize tumor cells to ICD, amplifying the immunostimulatory effects of OV-mediated lysis. The rationale for such combinations is that targeted therapies prime tumor cells for OV infection by suppressing antiviral pathways. OVs convert cytostatic responses into cytotoxic ones, ensuring tumor clearance rather than dormancy. Epigenetic modulators reprogram the tumor immunopeptidome, making tumors more visible to T cells activated by OV-induced antigen release. Thus, OV-targeted therapy combinations harness molecular vulnerabilities in signaling and epigenetic regulation to overcome resistance mechanisms, creating a synergistic immunovirological approach.

Although combining OVs with targeted or epigenetic therapies has emerged as a powerful approach to overcome tumor resistance and enhance immunogenicity, clinical translation remains inconsistent and mechanistically complex. Preclinical data provide compelling evidence of synergy; however, context-dependent signaling dynamics, variable tumor genetics, and antiviral feedback loops have limited reproducibility across tumor types and patient cohorts [122].

For example, several in vivo, ex vivo and clinical correlative studies focusing on combining BRAF inhibitors (vemurafenib or dabrafenib) or MEK inhibitors (trametinib) with OVs in melanoma and colorectal carcinoma models have shown that MAPK pathway suppression can indeed increase viral replication by downregulating IFN-stimulated genes (ISGs) such as OAS1, MX1, and IFIT1, rendering tumor cells more permissive to infection. In addition, BRAF blockade enhances melanoma antigen expression (MART-1, gp100, TYRP1), fostering improved T-cell recognition after OV-induced antigen release. Yet, durable synergy has not been consistent in clinical studies. Early trials combining T-VEC (HSV-1/GM-CSF) with BRAF/MEK inhibitors in metastatic melanoma demonstrated modest improvements in local response rates but no significant survival advantage, and in some cases, excessive local inflammation led to early discontinuation. Mechanistic follow-ups revealed that while MAPK inhibition transiently enhanced viral replication, compensatory activation of STAT1-driven IFN signaling and PD-L1 upregulation eventually reinstated antiviral and immunosuppressive barriers [123].

Similarly, combining EGFR inhibitors with OVs such as recombinant adenovirus (Ad5Δ24 or Ad5/3-Δ24) or reovirus (pelareorep) has produced mixed outcomes as reported by in vitro studies. While EGFR blockade can sensitize tumor cells to viral cytolysis by impairing Ras-dependent antiviral pathways, prolonged receptor inhibition can also suppress viral entry or replication in epithelial tumors reliant on EGFR for cellular uptake. For instance, pelareorep combined with erlotinib enhanced initial oncolysis in head-and-neck carcinoma models but reduced viral propagation over time due to impaired cell-to-cell spread [124].

Epigenetic modulators, including histone deacetylase inhibitors (HDACis) and DNA methyltransferase inhibitors (DNMTis), have shown similarly dual effects. On one hand, agents such as vorinostat, panobinostat, and valproic acid increase chromatin accessibility, reactivate silenced viral promoters, and boost replication of HSV, adenovirus, and vaccinia vectors. HDAC inhibition also induces expression of endogenous retroviral elements (ERVs), producing dsRNA intermediates that activate pattern recognition receptors (RIG-I, MDA5, TLR3) and promote ICD. On the other hand, sustained HDAC blockade can lead to excessive type I IFN release and upregulation of antiviral restriction factors (e.g., APOBEC3G, IFITM1), thereby reducing viral yield. Toxicity is another limitation: systemic HDACis or DNMTis can cause hematologic suppression, fatigue, and gastrointestinal toxicity, complicating their concurrent use with viral immunotherapy. These findings were validated across in vitro systems, rodent models, and human patients [125].

Clinical data remains sparse and heterogeneous. Trials evaluating pelareorep with HDACis or DNMTis in colorectal and pancreatic cancers have shown enhanced immune gene expression and occasional partial responses, but overall survival benefits remain minimal. A phase Ib trial combining decitabine with oncolytic vaccinia virus (JX-594/Pexa-Vec) demonstrated increased viral replication and immune activation markers (CXCL10, IFN-β) in patient biopsies, but without significant tumor regression. These mixed outcomes highlight that molecular context, tumor mutational load, baseline IFN tone, and epigenetic plasticity, dictates the balance between pro-viral and antiviral effects [126].

From a mechanistic standpoint, several factors underlie these inconsistencies. Targeted inhibitors can have pleiotropic effects on immune and viral pathways, sometimes enhancing replication while simultaneously upregulating antiviral cytokines that restrict infection. Epigenetic modulators, while reactivating latent viral promoters, can also reshape antigen presentation and immune composition in unpredictable ways, sometimes favoring immunogenicity, sometimes tolerance. Furthermore, optimal sequencing remains unresolved: whether targeted or epigenetic drugs should precede OV administration (to sensitize cells) or follow it (to sustain immune activation) depends on drug class, viral backbone, and tumor type [127].

Safety and resistance also present translational obstacles. The combined pro-inflammatory and cytotoxic activity of these agents can cause tumor necrosis, edema, and cytokine-release-like symptoms. In hepatocellular carcinoma models, for example, combining Pexa-Vec with sorafenib initially showed improved viral titers but later induced hepatic inflammation and transaminitis. In addition, the development of adaptive resistance—via reactivation of compensatory signaling pathways such as PI3K/AKT or JAK/STAT—can rapidly negate the sensitizing effects of targeted blockade [128].

#### 3.3.4. Chemotherapy/Radiotherapy

The integration of OVs with conventional cytotoxic modalities such as chemotherapy and radiotherapy provides bidirectional synergy at the molecular level. These combinations exploit tumor debulking, immune priming, and cellular stress responses that collectively enhance OV activity and antitumor immunity. Chemotherapeutics such as temozolomide (TMZ), cisplatin, or doxorubicin reduce tumor mass, thus decreasing interstitial pressure and improving OV penetration and distribution. Agents like cyclophosphamide and TMZ transiently suppress type I IFN responses and lymphocyte counts, reducing premature viral clearance and allowing more robust OV replication [129]. Drugs such as anthracyclines induce calreticulin exposure, HMGB1 release, and extracellular ATP secretion, all of which synergize with OV-mediated ICD to enhance DC activation and T cell priming [130]. Chemotherapeutics induce p53, ATM/ATR, and CHK1/CHK2 pathways, which increase tumor cell stress and make them more permissive to OV infection and lysis. On the other hand, ionizing radiation increases surface expression of receptors such as nectin-1 and CAR (coxsackievirus-adenovirus receptor), enhancing OV entry [131]. Radiation induces DNA double-strand breaks (DSBs), mitochondrial stress, and ER stress, leading to release of HMGB1, ATP, and other alarmins that amplify OV-triggered immune activation [132]. Radiation-derived cytosolic DNA activates cGAS-STING signaling, producing type I IFNs and CXCL10, which recruit effector T cells. In the presence of OVs, this creates a potent pro-inflammatory TME. Together, OV lysis and radiation-induced antigen release create a “dual antigenic burst,” driving strong CD8^+^ T cell priming and long-term memory formation. A landmark example is the combination of HSV G207 with radiotherapy in pediatric gliomas, where clinical trials demonstrated feasibility and safety, along with immune activation in the TME [133].

Although the integration of OVs with conventional cytotoxic therapies such as chemotherapy and radiotherapy represents one of the most mature and clinically translatable combination strategies, its success remains highly dependent on timing, dosage, and tumor context. While preclinical models have demonstrated potent synergy through immune activation and enhanced viral replication, clinical responses have been heterogeneous, reflecting the complexity of interactions between DNA damage, antiviral signaling, and immune modulation [134].

Mechanistically, the principal rationale for OV–chemotherapy synergy lies in the capacity of cytotoxic agents to lower antiviral defenses and increase tumor permissiveness to infection. Alkylating agents such as temozolomide (TMZ) or cyclophosphamide were demonstrated in vivo to transiently suppress type I IFN signaling and reduce circulating lymphocytes, thereby delaying immune-mediated viral clearance and allowing extended OV persistence. This strategy has been particularly relevant in glioblastoma multiforme (GBM) models, where TMZ combined with oncolytic adenoviruses (e.g., Ad5Δ24-RGD) or HSVs (HSV-G47Δ, HSV-1 G207) enhanced viral spread, apoptosis, and antitumor efficacy. However, this same immunosuppressive window can also dampen adaptive immune priming, potentially compromising the long-term immune memory that underlies durable tumor control [135].

Cytotoxic drugs also exert dose-dependent dual effects. Low-dose chemotherapy can induce ICD, characterized by calreticulin exposure, HMGB1 release, and ATP secretion, which synergizes with OV-mediated oncolysis to activate DCs and stimulate T-cell priming. Conversely, high-dose chemotherapy can destroy infiltrating immune effector cells, limiting the immune amplification required for viral-induced antitumor immunity. For example, combining pelareorep (reovirus) or VSV with doxorubicin or cisplatin enhanced local viral replication and ICD in preclinical breast and ovarian cancer models, but excessive cytotoxic dosing abrogated immune infiltration and delayed viral clearance. These observations underscore that therapeutic sequencing and dosing must be carefully optimized to preserve immune competence while maximizing viral oncolysis [136].

From a clinical standpoint, the evidence remains mixed. Trials evaluating pelareorep in combination with chemotherapeutics such as carboplatin–paclitaxel in ovarian and pancreatic cancers (e.g., NCT00998322, NCT01280058) demonstrated safety and modest improvements in progression-free survival but did not achieve statistically significant overall survival benefits. Similarly, a phase II trial combining Pexa-Vec (JX-594) with sorafenib or cisplatin-based regimens in hepatocellular carcinoma showed immune activation but limited tumor regression, suggesting that systemic chemotherapy may inadvertently impair viral propagation and immune recruitment. Notably, the HSV-1-derived G207 combined with radiotherapy in pediatric glioma demonstrated in vivo an encouraging safety, localized viral replication, and robust infiltration of CD8^+^ T cells and microglia, representing one of the first clear clinical proofs-of-concept for OV–radiotherapy synergy [137].

Radiotherapy presents similarly nuanced outcomes. Ionizing radiation increases OV infectivity by upregulating nectin-1, CAR, and other viral entry receptors, and by generating DNA double-strand breaks (DSBs) that release cytosolic DNA sensed by the cGAS–STING pathway, triggering type I IFN production and T-cell recruitment. In preclinical models, combining OVs such as reovirus, VSV, or adenovirus-Δ24 with radiation produced a “dual antigenic burst” that enhanced CD8^+^ T-cell priming and tumor clearance. However, excessive or fractionated radiation can paradoxically upregulate ISGs, activate STAT1, and reinforce antiviral resistance, thereby reducing viral replication. Moreover, radiation-induced hypoxia and fibrosis may create barriers to viral spread and limit subsequent immune infiltration [138].

Safety considerations remain a significant translational concern. Cytotoxic chemotherapy and radiation can increase susceptibility to infection, raising theoretical risks of uncontrolled viral replication or dissemination, particularly in immunocompromised patients. Although modern OV backbones (HSV-1 G207, Pexa-Vec, pelareorep) incorporate safety deletions that limit replication in normal tissues, patients with profound bone marrow suppression or prior irradiation may exhibit altered antiviral kinetics, requiring careful monitoring. Furthermore, overlapping toxicities—such as fatigue, cytopenias, mucositis, and radiation dermatitis—can complicate clinical management and mask early immune-related adverse events [139].

Mechanistically, another challenge lies in the temporal coordination of cytotoxic and viral therapies. Administering chemotherapy or radiation too early can prematurely eliminate infected cells, while delayed administration may fail to exploit the transient immunogenic milieu induced by viral infection. Optimal scheduling may thus require staggered protocols, where OVs first prime immune infiltration and antigen release, followed by low-dose chemotherapy or radiotherapy to amplify immune-mediated clearance. Early-phase trials combining pelareorep with radiotherapy in head and neck cancer or adenovirus with cisplatin–radiation in cervical carcinoma have supported this sequential model, demonstrating increased IFN-γ and CXCL10 signatures post-therapy [140].

#### 3.3.5. Neo-Adjuvant/Adjuvant Use

Administering OVs before surgery or primary therapy leverages their ability to reshape the tumor microenvironment (TME) and induce systemic immune priming. OV infection induces ER stress and ROS accumulation, leading to exposure of calreticulin (CRT) on the tumor cell surface, release of ATP, and nuclear translocation and release of HMGB1. These DAMPs engage PRRs (e.g., TLR4, P2RX7) on DCs, initiating antigen uptake and maturation. Tumor lysis releases a broad spectrum of TAAs and neoantigens, which are cross-presented via MHC class I and II to CD8^+^ and CD4^+^ T cells. This expands T cell specificity beyond the original tumor clone, reducing the chance of immune escape. Moreover, OV-induced chemokines (CXCL9, CXCL10, CCL5) recruit CXCR3^+^ effector T cells and NK cells, converting poorly infiltrated (“cold”) tumors into highly immunogenic (“hot”) tumors. Activated T cells migrate to lymph nodes, expand clonally, and can patrol peripheral tissues, enabling clearance of micrometastatic disease that may not yet be clinically detectable. This mechanism mimics a therapeutic vaccination effect initiated by the OV. When OVs are delivered after surgery or radiotherapy, the focus shifts to eliminating minimal residual disease (MRD) and preventing recurrence. Microscopic tumor nests left after surgery often upregulate stress ligands (e.g., NKG2D ligands) and downregulate MHC-I, making them more susceptible to NK-cell and OV-mediated killing. OV-mediated infection of residual tumor cells generates new waves of TAAs and DAMPs, enhancing antigen cross-presentation by DCs. This supports epitope spreading and broadens antitumor T cell immunity. Moreover, OVs can infect CTCs directly or stimulate type I IFN and proinflammatory cytokines (e.g., IL-12, TNF-α), activating NK cells to target CTCs in circulation. By establishing memory CD8^+^ T cells and memory NK cells, OVs provide durable immune protection against recurrence and metastatic relapse [123].

The neo-adjuvant and adjuvant application of OVs represents one of the most immunologically compelling yet clinically underexplored paradigms in immunovirotherapy. Conceptually, preoperative (neo-adjuvant) OV administration converts the primary tumor into an in situ vaccine, generating systemic immune activation that can target micrometastases or residual disease. Similarly, postoperative (adjuvant) OV delivery aims to eradicate minimal residual disease (MRD) and prevent recurrence. Despite a strong mechanistic rationale and promising preclinical data, the clinical translation of these strategies remains nascent, with limited trials, heterogeneous endpoints, and logistical barriers to integration into standard oncologic workflows [141].

In preclinical settings, several OVs—including HSV-1 (T-VEC and G47Δ), adenovirus (DNX-2401, ONCOS-102), and reovirus (pelareorep)—have demonstrated potent immunologic conversion of “cold” tumors into “hot” ones when used before surgery. For instance, in murine melanoma models, intratumoral T-VEC injection prior to resection led to a robust influx of CD8^+^ T cells, upregulation of CXCL9/CXCL10, and expansion of tumor-specific T-cell clones in draining lymph nodes. These responses were associated with reduced metastatic burden and improved survival after surgical excision. Similarly, in orthotopic glioma models, HSV-G207 and DNX-2401 administered pre-resection enhanced antigen presentation, delayed local recurrence, and extended survival, mimicking a vaccine-like “immune priming” effect. However, translating these findings to humans has been challenging due to tumor accessibility, timing constraints relative to surgery, and variability in patient immune competence [67].

Clinically, neo-adjuvant OV therapy remains in the early proof-of-concept stage. The most illustrative example is a small phase Ib trial in resectable melanoma, where intratumoral T-VEC administered before surgery led to elevated CD8^+^ and CD4^+^ T-cell infiltration, increased PD-L1 expression, and the induction of peripheral tumor-reactive T-cell clones. Importantly, approximately 30% of patients achieved a pathological complete response, and the immune profile of resected tumors suggested durable systemic immunity. Similar exploratory studies using pelareorep or Coxsackievirus A21 (Cavatak) prior to surgery in melanoma and bladder cancer confirmed localized immune activation but also revealed variability in viral spread, immune infiltration, and systemic cytokine induction. These findings highlight that while neo-adjuvant OVs can effectively ignite immune priming, consistent systemic protection and recurrence prevention remain uncertain [67].

Mechanistically, the dual immunologic nature of surgery poses additional complexity. While tumor removal reduces immunosuppressive load, surgical trauma itself induces a transient immunosuppressive and wound-healing phenotype, dominated by IL-10, TGF-β, and MDSC recruitment. This environment can hinder the immune activation that OVs seek to promote. Therefore, the timing of OV administration—either pre- or post-surgery—must be precisely optimized to exploit the inflammatory phase without exacerbating wound complications or immune exhaustion. Moreover, systemic antiviral immunity can limit re-infection after the initial dose, reducing the efficacy of repeated OV administrations during the perioperative window [142].

Safety and operational challenges also constrain widespread implementation. Perioperative OV delivery raises theoretical concerns regarding viral shedding, infection of surgical wounds, and nosocomial exposure, necessitating stringent biosafety protocols. Although clinical experience with T-VEC, G47Δ, and pelareorep has shown minimal shedding risk, the limited number of treated patients and the diversity of viral backbones preclude broad generalization. Furthermore, patient immune status profoundly influences outcomes: those receiving corticosteroids or neoadjuvant chemotherapy may exhibit impaired viral replication and dampened immune priming, reducing the therapeutic window for effective intervention [143].

## 4. Translational Integration and Clinical Realities in OV-Based Combination Strategies: Lessons from Clinical Experience

Despite compelling mechanistic rationale and extensive preclinical validation, the clinical translation of OV-based therapies has progressed more slowly and less consistently than initially anticipated. Although dozens of OV platforms have entered clinical testing over the past decades, the vast majority of programs have remained confined to phase I–II evaluation, with only a limited number advancing toward late-phase trials or regulatory approval [27,70,85,104,105,114,115,133,139,140]. Importantly, this pattern reflects not a lack of biological plausibility, but rather the emergence of recurrent translational bottlenecks that have constrained scalability, reproducibility, and durability of clinical benefit.

### 4.1. Lesson 1: Immune Activation Is Necessary but Insufficient

One of the most consistent lessons from OV clinical trials is that immune activation alone does not guarantee durable efficacy. OV-induced innate immune signaling, particularly IFN-I responses, promotes antigen presentation and immune priming but simultaneously drives adaptive immune resistance through PD-L1 upregulation on tumor and stromal cells [21,88,106,107]. This duality provided a strong rationale for OV–immune checkpoint inhibitor combinations, an approach supported by early-phase trials in melanoma, glioblastoma, and gastrointestinal malignancies [70,85,105,114,115,140]. However, clinical responses have been heterogeneous, and in many cases transient, underscoring that checkpoint blockade can unmask—but not fully overcome—pre-existing immunosuppressive programs. The lesson learned is that effective OV therapy requires not only immune activation, but sustained immune remodeling, including myeloid reprogramming, reversal of T cell exhaustion, and mitigation of compensatory inhibitory pathways.

### 4.2. Lesson 2: Preclinical Models Overestimate Viral Persistence and Immune Engagement

A major contributor to clinical attrition has been the discordance between preclinical systems and human disease. Murine models often permit robust OV replication, prolonged intratumoral persistence, and uniform immune engagement. In contrast, patients typically exhibit pre-existing antiviral immunity, rapid neutralizing antibody formation, and heterogeneous intratumoral viral distribution that severely restrict viral spread and transgene expression [66,67,90,139,141]. These factors compress the therapeutic window and reduce interpatient reproducibility. The lesson learned is that future translational pipelines must incorporate human-relevant models, including immunocompetent systems with prior viral exposure, ex vivo tumor explants, and early pharmacodynamic readouts that realistically capture viral kinetics in patients.

### 4.3. Lesson 3: Tumor Heterogeneity Dictates Clinical Responsiveness

Clinical experience has reinforced that OV efficacy is highly context-dependent. Variability in immune infiltration, antigen expression, stromal density, vascular permeability, and metabolic state leads to marked interpatient differences in OV infectivity and immune responsiveness [98,99,108,123]. Consequently, OV-based therapies have shown benefit only within specific biological niches, rather than across unselected patient populations.

Despite repeated calls for biomarker-driven stratification, validated predictive markers of OV responsiveness remain scarce, and their integration into trial design has been inconsistent [108,123,141]. The lesson learned is that OV trials must shift from broad eligibility criteria toward biomarker-informed patient selection, focusing on immune contexture, antiviral immunity, and tumor permissiveness rather than histology alone.

### 4.4. Lesson 4: Conventional Clinical Endpoints Underestimate OV Benefit

Another recurrent barrier has been trial design misalignment. OV therapies frequently induce delayed, mixed, or immune-mediated response patterns that are poorly captured by conventional RECIST criteria. In several trials, immune-mediated disease stabilization or pseudoprogression preceded clinical benefit, yet was interpreted as treatment failure, contributing to premature trial termination [70,85,105,115]. The lesson learned is that OV trials should adopt immune-adapted endpoints, extended evaluation windows, and correlative immune biomarkers to more accurately capture therapeutic activity and avoid false-negative outcomes.

### 4.5. Lesson 5: Manufacturing and Regulatory Complexity Limits Scalability

Beyond biological challenges, manufacturing, regulatory, and logistical constraints have significantly slowed clinical progression. OV-based therapeutics combine features of live viral agents, gene therapy vectors, and biologics, resulting in complex production pipelines and regulatory ambiguity [89,91,139]. Ensuring batch-to-batch consistency, genetic stability, and functional transgene expression—particularly for multi-armed platforms—remains technically demanding and costly, limiting scalability and commercial viability [141,142]. The lesson learned is that clinical success requires simplified, modular vector designs and early alignment with regulatory frameworks to ensure feasibility beyond proof-of-concept studies.

### 4.6. Lesson 6: Empirical Combination Strategies Are Insufficient

Although combination strategies have dominated OV clinical development, many regimens have been assembled empirically rather than mechanistically. In several cases, additive toxicity, insufficient synergy, or overlapping immune resistance mechanisms prevented sustained benefit [75,91,109,117,130,131,140,143]. The lesson learned is that OV combinations must be mechanism-informed, biomarker-guided, and temporally optimized, rather than based on parallel escalation of immunostimulatory agents.

### 4.7. Outlook: From Optimism to Precision Implementation

Collectively, these lessons emphasize that OV-based immunotherapy should not be regarded as a universally deployable or near-term solution. Instead, OVs function as context-dependent immunological catalysts, whose success depends on precise alignment between viral platform, tumor biology, immune landscape, and treatment sequencing [67,91,134,139,144]. Realistic expectations, adaptive trial designs, and improved translational modeling are therefore essential for advancing OV-based strategies toward sustainable clinical impact.

In summary, while OV-based therapies remain a promising component of the immuno-oncology arsenal, their successful clinical integration requires transparent acknowledgment of historical limitations, rigorous patient stratification, and rational trial design informed by both mechanistic insight and clinical experience. Addressing these lessons directly will be critical for translating OV-based immunotherapy from experimental promise into durable clinical benefit [140,141,142,143,144,145,146,147,148,149,150].

## 5. Clinical Applications and Key Trials

Several OV backbones have now reached regulated clinical use, and many additional virus families have been engineered as oncolytic platforms beyond those currently in late-phase trials. As summarized in Table 1, the landscape now includes HSV-1/HSV-2, adenoviruses, vaccinia/MVA, reovirus, poliovirus/other picornaviruses (Coxsackie A21, echoviruses, Seneca Valley Virus), paramyxoviruses (measles, NDV, Sendai), rhabdoviruses (VSV, Maraba MG1), parvoviruses (H-1PV), alphaviruses (Sindbis, SFV, VEE), orthomyxoviruses (influenza-engineered OVs), baculoviruses, borna virus-based constructs, and other emerging synthetic/synthetic-hybrid RNA virus chassis. Only a small subset of these platforms are in late clinical stages, but the breadth of the current pipeline demonstrates that OV immunotherapy has now evolved from a narrow “HSV/adenovirus” field to a broad inter-family synthetic virology discipline, where virtually any chassis with a safe replication profile and immunostimulatory phenotype can be converted into an OV. Three OVs are already approved in national markets (T-VEC in the United States, teserpaturev/G47Δ in Japan, and H101 in China), and approximately thirty additional derived backbones are in human trials across North America, Europe, and Asia (Table 1).

### 5.1. Talimogene Laherparepvec (T-VEC; HSV-1/GM-CSF)

First FDA-approved OV (2015) for unresectable cutaneous, subcutaneous, and nodal melanoma (OPTiM trial). Although early-phase studies suggested enhanced activity in combination with pembrolizumab, the global phase III MASTERKEY-265/KEYNOTE-034 trial did not improve PFS or OS versus pembrolizumab alone [151,152]. Nevertheless, neoadjuvant T-VEC, intratumoral T-VEC + intratumoral immunomodulators, and T-VEC + next generation co-stimulation agonists remain active avenues of development (Table 1).

### 5.2. Teserpaturev/G47Δ (Third-Generation HSV-1; “Delytact”)

G47Δ harbors triple genetic alterations—deletions in ICP34.5 and ICP47, plus inactivation of ICP6—combined with promoter rewiring. These engineered changes enhance tumor selectivity, restrict replication to malignant cells with high nucleotide pools, and increase intratumoral immunogenicity. In Japan, a Phase II trial in recurrent/residual glioblastoma reported improved survival outcomes compared with historical controls, which led to conditional national approval in 2021 [153]. Multiple next-generation constructs based on the G47Δ backbone—including fourth-generation vectors armed with cytokines and co-stimulatory molecules—are now being applied in intracranial settings and early-phase clinical testing (Table 1).

### 5.3. H101 (Oncorine; Adenovirus)

H101 was approved in China in 2005, the world’s first marketed OV, for use with chemotherapy in nasopharyngeal/head & neck cancer. Modern derivatives (e.g., Ad5F35, hTERT-Ad5 variants, payloaded Ad5 backbones) continue to be evaluated across tumor types, often in combination with PD-1 blockade or radiotherapy [154] (Table 1).

### 5.4. Other Clinically Advanced Exemplary

**Adenovirus (OBP-301/Telomelysin).** hTERT-driven Ad5 used in gastric and esophageal cancers, including ongoing pembrolizumab and chemoradiation combinations [155].

**Vaccinia (Pexa-Vec, Olvi-Vec, TG6002, MVA-Fcu1).** Olvi-Vec showed encouraging results in ovarian cancer chemo-priming, whereas Pexa-Vec failed in phase III HCC (PHOCUS) [156], and newer vaccinia constructs now rely on heavy payloading (GM-CSF, FCU1 suicide genes, chemokines).

**Reovirus (pelareorep).** FDA Fast Track; widely combined with ICIs and chemotherapy in breast and GI disease [157].

**Picornavirus platforms.** PVSRIPO in recurrent glioblastoma demonstrated long-tail survival (~20% survival beyond 2–3 years); Coxsackievirus A21 (CAVATAK) and SVV-001 (Seneca Valley Virus) continue to build neuroendocrine/ICAM-1 directed programs [158].

**Measles virus/NDV/Sendai.** MV-NIS and MV-CEA enable noninvasive imaging and are under evaluation in multiple myeloma/ovarian cancer [159]; NDV/Sendai retain strong natural immunogenicity.

**Rhabdoviruses (VSV-IFNβ, Maraba MG1).** VSV-IFNβ constructs are in phase I and show strong type I IFN-shaped innate activation, with clear evidence of antigen spread in melanoma/hematologic cancers [160]; Maraba MG1 is being developed as OV prime + viral vector vaccine boost (Table 1).

## 6. Challenges, Limitations and Future Directions

Despite promising results, several challenges hinder the widespread clinical success of OVs.

### 6.1. Challenges and Limitations

#### 6.1.1. Delivery and Biodistribution

Systemic delivery of OVs faces hurdles such as rapid clearance by the reticuloendothelial system, neutralizing antibodies (NAbs), and physical barriers within solid tumors. Intratumoral delivery, while effective locally, is limited to accessible lesions. Strategies to improve delivery include:

- *Physical Methods:* Ultrasound-mediated cavitation, convection-enhanced delivery (CED) for brain tumors.
- *Chemical/Physical Modifications:* PEGylation or coating with polymers to reduce immunogenicity and increase circulation time; conjugation to cell-penetrating peptides or targeting ligands.
- *Biological Carriers:* Encapsulation in cells (e.g., mesenchymal stem cells, carrier cells), liposomes, or nanoparticles. Cell carriers can potentially home to tumors and protect the virus from immune clearance. Nanovesicles are an emerging platform.
- *Overcoming NAbs:* Albumin-binding viruses or encapsulation techniques, use of less prevalent serotypes or engineering viruses to avoid common neutralizing epitopes, and chemical shielding.
- *Routes of Administration:* Exploring different routes like intravenous, intraperitoneal, or intravesicular delivery for tumor-specific applications.

#### 6.1.2. Tumor Heterogeneity and Resistance

Intratumor heterogeneity may generate subpopulations of cells resistant to OV infection or lysis. The immune suppressive TME can also limit the effectiveness of OV-induced immune responses. Strategies to overcome resistance include using cocktails of different OVs or combining OVs with agents that modulate the TME (e.g., PI3Kδ inhibitors).

#### 6.1.3. Patient Selection and Biomarkers

Accumulating clinical experience strongly suggests that improved patient selection is critical for the future success of oncolytic virotherapy. Rather than treating OVs as broadly applicable agents, emerging evidence supports a precision-based approach guided by tumor permissiveness and immune contexture. Candidate biomarkers under investigation include baseline immune infiltration (e.g., CD8^+^ T cells, dendritic cells), interferon signaling signatures, antigen presentation capacity (MHC-I, β2-microglobulin), and expression of viral entry receptors. In parallel, host-related factors such as antiviral serostatus, innate immune responsiveness, and prior viral exposure may influence therapeutic window and durability of response. Functional biomarkers may also play an important role. Early intratumoral viral replication, induction of immunogenic cell death markers, and dynamic changes in cytokine or chemokine profiles following OV administration could serve as on-treatment indicators of responsiveness. Incorporation of such biomarkers into adaptive trial designs may enable real-time treatment optimization, rational combination selection, and early discontinuation in non-responders. Looking forward, integration of multi-parameter biomarker panels, spatial immune profiling, and longitudinal immune monitoring is likely to be essential for identifying patients most likely to benefit and for directing OV-based therapies toward sustainable clinical impact.

#### 6.1.4. Balancing Antiviral and Antitumor Immune Responses

**Figure 3:**
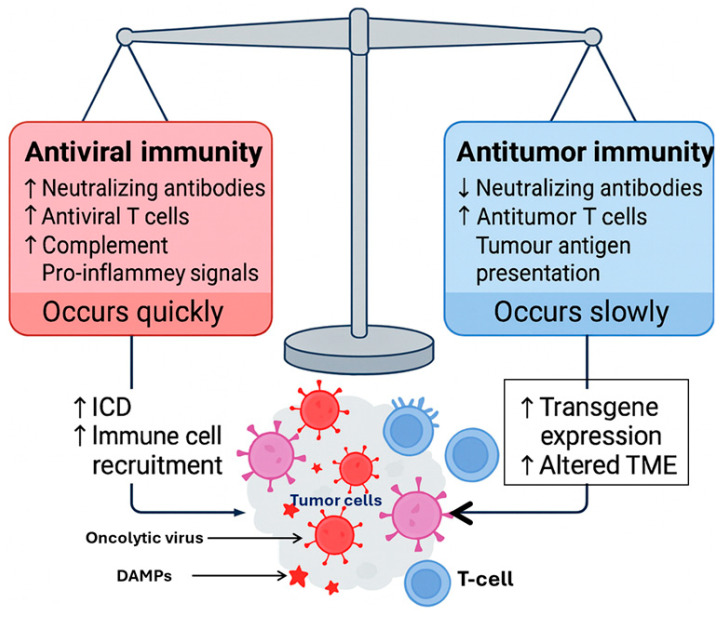
Balance between antiviral and antitumor immunity following OV administration. The illustration depicts the immunological trade-off induced by OVs, represented as a balance between rapid antiviral immunity (**left**) and slower-developing antitumor immunity (**right**). Antiviral responses occur quickly and include increased neutralizing antibodies, antiviral T-cell activation, complement activity, and pro-inflammatory signaling. These processes enhance ICD and immune-cell recruitment but can limit viral persistence. In contrast, antitumor immunity develops gradually, characterized by reduced antiviral neutralization, increased antitumor T-cell activity, and improved tumor-antigen presentation. This arm supports enhanced transgene expression within the tumor and promotes remodeling of the TME. Together, these opposing forces determine the therapeutic outcome of OV-mediated cancer immunotherapy.

A major limitation of oncolytic virotherapy is that antiviral immune responses overwhelmingly dominate antitumor immunity, often preventing the development of the robust, durable T-cell responses needed for tumor eradication (Figure 3). This dominance begins within minutes of viral entry into the tumor. Tumor and stromal cells rapidly detect viral nucleic acids through pattern-recognition receptors such as cGAS–STING, RIG-I, MDA5, and endosomal TLR3. Activation of these sensors triggers a potent IFN-I cascade that induces hundreds of IFN-stimulated genes (ISGs), including PKR, OAS, and MxA. These ISGs suppress viral transcription and translation, disrupt viral genome replication, and induce apoptosis of infected cells. Because this antiviral program unfolds far more rapidly than antigen processing and cross-presentation, many OV-infected tumor cells undergo premature death before they can complete sufficient replication cycles to release TAAs or DAMPs. As a result, the quantity and diversity of antigens available for DC priming is drastically reduced, limiting the development of a strong antitumor T-cell repertoire [161].

In parallel, IFN-I–induced chemokines, particularly CXCL9 and CXCL10, drive the rapid infiltration of natural killer (NK) cells into the TME. NK cells recognize OV-infected tumor cells through NKG2D ligands and missing-self signals, enabling them to kill infected cells well before adaptive immune responses emerge. While this is advantageous for eliminating viral infections in natural settings, in the context of virotherapy it prematurely destroys the very “in situ vaccine factories” needed to generate ICD and broad antigen release. As NK cell-mediated cytotoxicity restricts viral propagation, the potential for epitope spreading and sustained antitumor immune priming is significantly diminished. Consequently, the immune landscape becomes heavily skewed toward clearing the virus rather than identifying and eliminating tumor cells.

Moreover, DCs exposed to this intense antiviral environment preferentially internalize and present viral antigens, which are abundant and highly immunogenic compared to the limited pool of tumor antigens released. This results in robust expansion of antiviral CD8^+^ T cells, which rapidly outcompete tumor-specific T cells for cytokines, co-stimulation, and access to APCs. High-affinity TCR–viral peptide interactions generate potent effector responses, whereas tumor-specific TCRs, which typically recognize lower-affinity neoantigens, receive insufficient activation signals to expand effectively. This competitive imbalance further entrenches antiviral dominance and suppresses the generation of durable antitumor immunity [162,163].

Adding to this complexity, IFN-I signaling upregulates several immunosuppressive pathways that disproportionately inhibit tumor-specific T cells. Infected tumor cells and local myeloid populations markedly increase expression of PD-L1, IDO, and other checkpoint molecules, while T cells upregulate exhaustion markers such as PD-1, TIM-3, and LAG-3. Although antiviral T cells often continue to function due to strong antigenic stimulation, tumor-specific T cells, already receiving weaker activation signals, are rapidly driven into exhaustion or anergy. Thus, antiviral inflammation unintentionally creates a TME hostile to the expansion and persistence of tumor-reactive lymphocytes [162,163].

Finally, within days of initial infection, the host mounts a robust humoral antiviral response characterized by the production of neutralizing antibodies and complement activation. These antibodies rapidly eliminate circulating virus and significantly curtail reinfection of tumor cells upon subsequent OV administrations. By terminating ongoing cycles of oncolysis, neutralizing antibodies sharply limit the sustained antigen release, DC activation, and epitope spreading that are essential for durable antitumor immunity. Consequently, the therapy’s immunogenic boost becomes transient, and the cancer escapes immune surveillance once antiviral clearance is complete.

Collectively, these mechanisms illustrate that antiviral immunity—fast, potent, and evolutionarily prioritized—can overshadow, suppress, or prematurely abort the antitumor immune responses that oncolytic virotherapy aims to generate. Overcoming this hierarchy remains one of the central challenges in designing next-generation OVs capable of balancing antiviral clearance with effective and sustained tumor-specific immunity [162,163].

#### 6.1.5. Safety and Toxicity

While generally well-tolerated, OVs can cause inflammation, fever, and, rarely, more severe immune-related adverse events. Careful monitoring is essential. The risk of viral dissemination or recombination (though low with replication-deficient vectors) needs consideration.

#### 6.1.6. Manufacturing and Regulatory Hurdles

The production clinical-grade OVs is technically complex and costly. Regulatory approval requires careful navigation for novel engineered agents, especially regarding defining the product and ensuring consistency.

### 6.2. Future Directions

- Biomarkers: Development predictive biomarkers (viral receptors, immune status, microbiome composition) to identify patients most likely to benefit from specific OV treatments.
- Personalized OV Therapy: Tailoring OVs based on tumor molecular profiling and patient-specific features such as specific receptor expression and immune status.
- Advanced Engineering: Creating “smart” viruses with enhanced tumor targeting, controlled replication (e.g., using miRNA targets for tissue specificity), and sophisticated payload release mechanisms (e.g., inducible promoters). Expanding the range of payloads, including gene editing tools like CRISPR, is also an area of interest. Synthetic virology approaches are emerging.
- Improved Combination Strategies: Identifying optimal combinations and sequencing of OVs with ICIs, ACTs, targeted therapies, and conventional therapies. Understanding biomarkers predictive of response to specific combinations is crucial.
- Novel Delivery Systems: Continued innovation in delivery methods, including image-guided delivery and responsive nanocarriers. Exploring routes like inhalation for lung cancers or intraperitoneal delivery for peritoneal carcinomatosis is also important.
- Integration with Microbiome Research: Investigating the interactions between OVs, the tumor microbiome, and the host’s gut microbiome, and how these interactions influence treatment efficacy.
- Virus Cocktails: Using combinations of different OVs to potentially overcome resistance and target multiple pathways.

## 7. Conclusions

Oncolytic viruses (OVs) have emerged as a compelling experimental platform at the intersection of virology, molecular biology, and immuno-oncology, offering the dual capacity to induce direct tumor cell lysis and modulate the tumor microenvironment (TME) toward immune activation. Clinical milestones such as the approval of talimogene laherparepvec (T-VEC) and G47Δ demonstrate that OV-based therapies are clinically feasible under specific conditions. However, accumulated clinical experience over the past decades also makes clear that broad and rapid clinical implementation has proven far more challenging than initially anticipated.

Despite extensive preclinical promise and numerous early-phase clinical trials, the majority of OV programs have not progressed beyond phase I–II evaluation. This limited advancement reflects persistent barriers rather than a lack of biological rationale. Tumor heterogeneity, pre-existing and treatment-induced antiviral immunity, restricted intratumoral viral spread, and variability in immune responsiveness continue to undermine reproducibility and durability of clinical benefit. In addition, challenges related to systemic delivery, manufacturing scalability, regulatory complexity, and endpoint selection have further constrained late-stage development. These realities underscore that OV therapy cannot currently be regarded as a universally applicable or near-term mainstream treatment.

Advances in molecular engineering—including promoter targeting, payload optimization, and miRNA-based regulation—have improved selectivity and safety, but have not fully overcome the fundamental biological and translational constraints observed in patients. Similarly, emerging delivery strategies such as cellular carriers, nanocarriers, and image-guided administration offer incremental improvements rather than definitive solutions. As such, future progress is likely to depend less on further escalation of viral complexity and more on precision implementation, including biomarker-guided patient selection, rational combination strategies, and careful treatment sequencing aligned with immune competence.

Looking forward, OVs should be viewed not as stand-alone curative agents, but as context-dependent immunological tools whose effectiveness depends on precise alignment between viral platform, tumor biology, immune landscape, and clinical design. Integration into precision oncology frameworks—supported by predictive biomarkers, adaptive trial designs, and realistic clinical endpoints—will be essential to determine where and for whom OV-based therapies can deliver meaningful benefit.

In summary, while oncolytic virotherapy remains an important and evolving component of the immuno-oncology arsenal, its future clinical impact will depend on transparent recognition of past limitations, rigorous translational discipline, and restrained expectations. Progress toward durable clinical success is likely to be incremental rather than revolutionary, but continued refinement grounded in clinical reality may ultimately define a sustainable role for OVs in selected patient populations with unmet therapeutic needs.

## Figures and Tables

**Table 1:** Virus Families and Engineered Oncolytic Virus (OV) Platforms Used in Oncolytic Immunotherapy.

Virus Family	Representative OV Platforms	Key Engineering Strategies	Cancer Targets (Examples)	Clinical Status	Primary Barrier to Clinical Efficacy
**A. Clinically Advanced Oncolytic Virus Platforms (Human Testing)**					
Herpesviridae	T-VEC; G47Δ; HF10; NV1020; RP1/RP2/RP3; oHSV-IL12	ICP34.5/ICP47 deletions; GM-CSF or IL-12 arming; antibody/minibody payloads; tumor-specific promoters	Melanoma; GBM; liver metastases; pancreatic and other solid tumors	FDA approval (T-VEC); Japan approval (G47Δ); phase I–III trials	Limited intratumoral spread; pre-existing HSV immunity; local toxicity
Adenoviridae	H101; ONYX-015; DNX-2401; OBP-301; Enadenotucirev; LOAd703	E1A/E1B deletions; hTERT promoters; RGD retargeting; CD40L/4-1BBL and cytokine arming	HNSCC; GBM; ovarian; pancreatic; colorectal; liver tumors	H101 approved in China; global phase I–II trials	Pre-existing adenoviral immunity; inflammatory toxicity
Poxviridae (Vaccinia/MVA)	Pexa-Vec; Olvi-Vec; TG6002; JX-963	TK deletion; GM-CSF/IFN payloads; suicide genes; systemic replication	HCC; melanoma; colorectal liver metastases	Phase I–III trials	Neutralizing antibodies; safety in immunocompromised hosts
Reoviridae	Pelareorep (Reolysin)	Natural Ras-pathway tropism; ICD induction; combination with ICIs	Breast; GI cancers; melanoma; lymphoma	FDA Fast Track; phase II–III trials	Modest single-agent efficacy; reliance on combinations
Picornaviridae	PVSRIPO; CAVATAK (CVA-21); SVV-001	Receptor tropism (CD155, ICAM-1); IRES rewiring	GBM; melanoma; SCLC	Phase I–II trials	Neurotoxicity risk; narrow tumor tropism
Paramyxoviridae	MV-NIS; MV-CEA; NDV-PV701	Receptor retargeting; cytokine arming; strong innate activation	Myeloma; ovarian; lung; PDAC; bladder	Phase I–II trials	Antiviral immunity; limited persistence
Rhabdoviridae	VSV-IFNβ; MG1 (Maraba)	IFN-β safety switch; NIS imaging; in situ vaccination	Melanoma; AML; TNBC	Early clinical trials	Rapid antiviral clearance; neurotoxicity concerns
Parvoviridae	H-1PV (ParvOryx)	Natural oncotropism; NS1-mediated apoptosis	GBM; PDAC	Phase I–II trials	Limited replication efficiency
**B. Emerging and Preclinical Oncolytic Virus Platforms**					
Retroviridae	RRV-CD; RRV-TK; MLV-based vectors	Pro-drug converting enzymes; stable integration	Glioma; liver metastases	Phase I–II	Insertional mutagenesis risk; slow kinetics
Orthomyxoviridae	NS1-deleted influenza OVs	Enhanced IFN induction; respiratory targeting	Lung; ovarian; colon	Preclinical–early clinical	Safety concerns; host immunity
Bornaviridae	Recombinant BDV OVs	Low-cytolytic persistent infection	CNS tumors	Preclinical	Risk of viral persistence
Baculoviridae	Baculo-GM-CSF; Baculo-STING	Large payload capacity; non-replicating in mammals	Hepatobiliary; PDAC	Preclinical	Lack of replication limits efficacy
Alphaviridae	SFV; Sindbis; VEE OVs	High-level expression; strong innate immunity	Breast; ovarian; glioma	Preclinical–early-phase I	Systemic toxicity; rapid clearance
Coronaviridae	Engineered MHV-1	Synthetic chassis; innate immune activation	Experimental tumor models	Preclinical	Biosafety and regulatory constraints
Flaviviridae	Zika-based OVs	Neural progenitor tropism; IFN-sensitive attenuation	GBM; pediatric brain tumors	Preclinical	Neurotoxicity risk
Togaviridae	Ross River virus OVs	Rapid replication; antigen expression	Solid tumors	Preclinical	Limited translational experience
Bunyavirales	Attenuated RVFV OVs	Strong RIG-I/MDA5 activation	Liver cancers	Preclinical	Hepatotoxicity and safety concerns

## Data Availability

No data are associated with the manuscript.

## Abbreviations

**ACT**	Adoptive Cell Therapy
**ADCC**	Antibody-Dependent Cellular Cytotoxicity
**ADCP**	Antibody-Dependent Cellular Phagocytosis
**AKT**	Protein Kinase B
**APC**	Antigen-Presenting Cell
**ATM**	Ataxia Telangiectasia Mutated
**ATP**	Adenosine Triphosphate
**ATR**	Ataxia Telangiectasia and Rad3-Related
**BRAF**	v-Raf Murine Sarcoma Viral Oncogene Homolog B
**CAR**	Chimeric Antigen Receptor
**CEA**	Carcinoembryonic Antigen
**CED**	Convection-Enhanced Delivery
**CMV**	Cytomegalovirus
**CRISPR**	Clustered Regularly Interspaced Short Palindromic Repeats
**CRS**	Cytokine Release Syndrome
**CRT**	Calreticulin
**CSF**	Colony-Stimulating Factor
**CTL**	Cytotoxic T Lymphocyte
**CTLA-4**	Cytotoxic T-Lymphocyte-Associated Protein 4
**DAMP**	Damage-Associated Molecular Pattern
**DC**	Dendritic Cell
**DNA**	Deoxyribonucleic Acid
**DNMT**	DNA Methyltransferase
**DNX-2401**	Delta-24-RGD Oncolytic Adenovirus
**ECM**	Extracellular Matrix
**EGFR**	Epidermal Growth Factor Receptor
**ERK**	Extracellular Signal-Regulated Kinase
**ERV**	Endogenous Retrovirus
**FDA**	Food and Drug Administration
**GBM**	Glioblastoma Multiforme
**GM-CSF**	Granulocyte-Macrophage Colony-Stimulating Factor
**HDAC**	Histone Deacetylase
**HIF**	Hypoxia-Inducible Factor
**HSV**	Herpes Simplex Virus
**ICD**	Immunogenic Cell Death
**IDO**	Indoleamine 2,3-Dioxygenase
**IFN**	Interferon
**IL**	Interleukin
**IRF**	Interferon Regulatory Factor
**JAK**	Janus Kinase
**LAG-3**	Lymphocyte-Activation Gene 3
**MDSC**	Myeloid-Derived Suppressor Cell
**MHC**	Major Histocompatibility Complex
**miRNA**	MicroRNA
**NF-κB**	Nuclear Factor Kappa B
**NK**	Natural Killer Cell
**NDV**	Newcastle Disease Virus
**OV**	Oncolytic Virus
**PD-1**	Programmed Cell Death Protein 1
**PD-L1**	Programmed Death-Ligand 1
**PRR**	Pattern Recognition Receptor
**RECIST**	Response Evaluation Criteria in Solid Tumors
**RNA**	Ribonucleic Acid
**scFv**	Single-Chain Variable Fragment
**STING**	Stimulator of Interferon Genes
**TAA**	Tumor-Associated Antigen
**TAM**	Tumor-Associated Macrophage
**TCR**	T-Cell Receptor
**TIGIT**	T Cell Immunoreceptor with Ig and ITIM Domains
**TLS**	Tertiary Lymphoid Structure
**TME**	Tumor Microenvironment
**TNF**	Tumor Necrosis Factor
**TriTE**	Trispecific T-Cell Engager
**VISTA**	V-Domain Ig Suppressor of T-Cell Activation
**VSV**	Vesicular Stomatitis Virus
